# PpBBX32 and PpZAT5 modulate temperature-dependent and tissue-specific anthocyanin accumulation in peach fruit

**DOI:** 10.1093/hr/uhae212

**Published:** 2024-07-30

**Authors:** Dan Huang, Lei Xue, Yueqin Lu, Mengfei Liu, Kui Lin-Wang, Andrew C Allan, Bo Zhang, Kunsong Chen, Changjie Xu

**Affiliations:** Zhejiang Key Laboratory of Horticultural Crop Quality Improvement, Zhejiang University Zijingang Campus, Hangzhou, PR China; Zhejiang Key Laboratory of Horticultural Crop Quality Improvement, Zhejiang University Zijingang Campus, Hangzhou, PR China; The State Agriculture Ministry Laboratory of Horticultural Plant Crop Growth and Development, Zhejiang University Zijingang Campus, Hangzhou, PR China; The State Agriculture Ministry Laboratory of Horticultural Plant Crop Growth and Development, Zhejiang University Zijingang Campus, Hangzhou, PR China; New Cultivar Innovation, the New Zealand Institute for Plant & Food Research Limited (Plant & Food Research) Mt Albert, Auckland, New Zealand; New Cultivar Innovation, the New Zealand Institute for Plant & Food Research Limited (Plant & Food Research) Mt Albert, Auckland, New Zealand; School of Biological Sciences University of Auckland, Private Bag, Auckland, New Zealand; Zhejiang Key Laboratory of Horticultural Crop Quality Improvement, Zhejiang University Zijingang Campus, Hangzhou, PR China; The State Agriculture Ministry Laboratory of Horticultural Plant Crop Growth and Development, Zhejiang University Zijingang Campus, Hangzhou, PR China; Zhejiang Key Laboratory of Horticultural Crop Quality Improvement, Zhejiang University Zijingang Campus, Hangzhou, PR China; The State Agriculture Ministry Laboratory of Horticultural Plant Crop Growth and Development, Zhejiang University Zijingang Campus, Hangzhou, PR China; Zhejiang Key Laboratory of Horticultural Crop Quality Improvement, Zhejiang University Zijingang Campus, Hangzhou, PR China; The State Agriculture Ministry Laboratory of Horticultural Plant Crop Growth and Development, Zhejiang University Zijingang Campus, Hangzhou, PR China

## Abstract

Anthocyanins are important compounds for fruit quality and nutrition. The R2R3 MYB transcription factor PpMYB10.1 is known to be critical for regulating anthocyanin accumulation in peach. However, regulatory factors upstream of *PpMYB10.1* which control temperature-dependent, cultivar-contrasted and tissue-specific anthocyanin accumulation remain to be determined. In this study, differential anthocyanin accumulation in the outer flesh near the peel (OF) of peach [*Prunus persica* (L.) Batsch] was observed between cultivars ‘Zhonghuashoutao’ and ‘Dongxuemi’, as well as among different storage temperatures and different fruit tissues of ‘Zhonghuashoutao’. By cross-comparisons of RNA-Seq data of samples with differential anthocyanin accumulation, transcription factor genes *PpBBX32* and *PpZAT5* were identified. These were functionally characterized as two positive regulators for anthocyanin accumulation via transient expression and genetic transformation. Various interaction assays revealed that both PpBBX32 and PpZAT5 can directly activate the *PpMYB10.1* promoter and meanwhile interact at protein level as a PpZAT5-PpBBX32-PpMYB10.1 complex. Furthermore, the results of *in silico* analysis and exogenous application of methyl jasmonate (MeJA) indicated that MeJA favored anthocyanin accumulation, while it was also found that anthocyanin accumulation as well as *PpBBX32* and *PpZAT5* expression correlated significantly with endogenous JA and JA-Ile in different fruit tissues. In summary, PpBBX32 and PpZAT5 are upstream activators of *PpMYB10.1*, allowing JAs to take part in temperature-dependent and tissue-specific anthocyanin accumulation by modulating their expression. This work enriches the knowledge of the transcriptional regulatory mechanisms for differential anthocyanin accumulation under internal and external factors.

## Introduction

Anthocyanins, a class of crucial pigments in plants, are responsible for providing tissues with hues of red, blue, or purple [1, 2]. In plants, they play a number of vital biological roles, including attracting animals to pollinate and disperse seeds, as well as enhancing resistance to various abiotic and biotic stresses [1, 2]. Additionally, anthocyanins confer various health benefits for animals and humans, including anti-aging effects, antithrombotic activities and cancer prevention [3].

Researchers have found that anthocyanin biosynthesis exhibits high conservation among plant species via the phenylpropanoid and flavonoid pathways where a series of enzymes, including phenylalanine ammonia lyase (PAL), chalcone synthase (CHS), chalcone isomerase (CHI), flavanone 3-hydroxylase (F3H), flavonoid 3′-hydroxylase (F3’H), flavonoid 3′,5′-hydroxylase (F3’5’H), dihydroflavonol 4-reductase (DFR), leucoanthocyanidin dioxygenase/anthocyanidin synthase (LDOX/ANS), and (UDP)-glucose:flavonoid-3-*O*-glycosyltransferase (UFGT), are involved [4–6]. The synthesized anthocyanins are then transported to the vacuole via transporters, especially glutathione S-transferase (GST) [7]. The biosynthesis as well as the transport of anthocyanins were transcriptionally regulated by a MYB-bHLH-WDR (MBW) transcriptional regulatory complex comprising of a DNA-binding R2R3 MYB transcription factor (TF), a MYC-like basic helix–loop–helix (bHLH) TF and a WD40-repeat protein [2, 8, 9].

Anthocyanin biosynthesis is affected by various environmental factors, especially light and temperature [10, 11]. Light is a crucial factor in the regulation of anthocyanin accumulation, which has been extensively studied and was found that blue and UV light are generally most effective [12, 13]. Anthocyanin accumulation is also strongly influenced by another significant environmental factor, temperature, where moderately lower temperatures generally facilitate the production of anthocyanins [14–16]. Anthocyanin accumulation is also regulated, positively or negatively, by plant hormones as well as other unrevealed genetic or developmental factors [17, 18].

The regulation of anthocyanin accumulation is sometimes not directly fulfilled via influencing the expression of MBW members, but mediated by a number of positive or negative transcription factors, such as MADS-box protein, basic region/leucine zipper (bZIP), B-box protein (BBX), NAC and WRKY, upstream of anthocyanin related critical MYB genes [11]. For example, in ‘Red Zaosu’ pear, PpyBBX16 induces *PpyMYB10* expression to promote anthocyanin accumulation under light [19]; while PpyERF9 suppresses the expression of *PpyRAP2.4* and *PpyMYB114* through histone deacetylation, thereby inhibiting ethylene-induced anthocyanin biosynthesis [20]. With the assistance of MdbZIP23, MdNAC1 activates the transcription of *MdMYB10* and mediates ABA induced anthocyanin accumulation in ‘Fuji’ apple [21].

Peach (*Prunus persica*) is a globally important fruit crop with peel and flesh color being crucial for its commercial value. The red pigment in ripe peach fruit is the anthocyanin cyanidin-3-*O*-glucoside (C3G), which is synthesized in a pathway conserved in plants [22]. After synthesis, mediated by *PpGST1*, anthocyanin is transported into vacuoles for storage [7]. The main transcription factor responsible for modulating expression of genes related to anthocyanin biosynthesis and transport is PpMYB10.1, with PpbHLH3 and PpWD40–1 serving as partners to form the MBW complex [23, 24]. PpMYB10.1 plays a crucial role in regulating the differential or contrasted anthocyanin accumulation in peach as affected by internal factors such as genotype, plant hormones, sugars and external stimuli like light and temperature [10, 11]. Additionally, there is a differential anthocyanin accumulation between peel (P), outer flesh near the peel (OF), and inner flesh around the stone (IF) in peach fruit [7]. In a blood-fleshed peach, PpNAC1, a positive regulator, interacts with another NAC TF named BLOOD (BL) to activate the *PpMYB10.1* promoter, which promotes anthocyanin accumulation [25]. However, in different cultivars, there may be alternative reasons contributing to the accumulation of anthocyanin. Anthocyanin biosynthesis in the IF is also driven by PpMYB10.1, which stimulates downstream genes including the transporter *PpGST1* [7, 26], but regulatory factors upstream of *PpMYB10.1* are unknown. In ‘Hujingmilu’ peach, ELONGATED HYPOCOTYL 5 (HY5), a key component in the light signal transduction cascade, is a transcription activator of itself as well as *PpMYB10.1* and anthocyanin biosynthetic genes, thus mediating ultraviolet light induced anthocyanin accumulation in peel [7, 13, 24]. In the peach cultivar ‘Zhonghuashoutao’ (‘ZHST’), fruit stored at 16°C accumulated anthocyanin in the OF as a result of strong expression of *PpMYB10.1*, but not at 12°C or lower [16]. In summary, the cultivar-contrasted, tissue-specific and temperature-dependent accumulation of anthocyanin in peach is closely associated with the expression of *PpMYB10.1*. The regulatory factors upstream of *PpMYB10.1* and the in-depth mechanisms underlying the differential anthocyanin accumulation are an area of active research.

This study aimed to identify the upstream TFs regulating *PpMYB10.1* and to further explore possible links between their expression and endogenous/external stimuli. Through comparison of phenotype and gene expression in different fruit tissues, cultivars or at different storage temperatures, we identified and functionally characterized PpBBX32 and PpZAT5, proteins homologous to Arabidopsis B-box protein 32 and ZINC FINGER of *A. thaliana* 5, respectively, as activators upstream of *PpMYB10.1.* It was also found that the expression of these two TFs is stimulated by methyl jasmonate (MeJA) treatment and is well correlated with levels of endogenous jasmonates (JAs) in different tissues. The study provides novel insights into the specific mechanisms underlying temperature-dependent and tissue-specific anthocyanin accumulation in peach fruit.

## Results

### Temperature-dependent, cultivar-contrasted and tissue-specific anthocyanin accumulation in peach fruit

**Figure 1:**
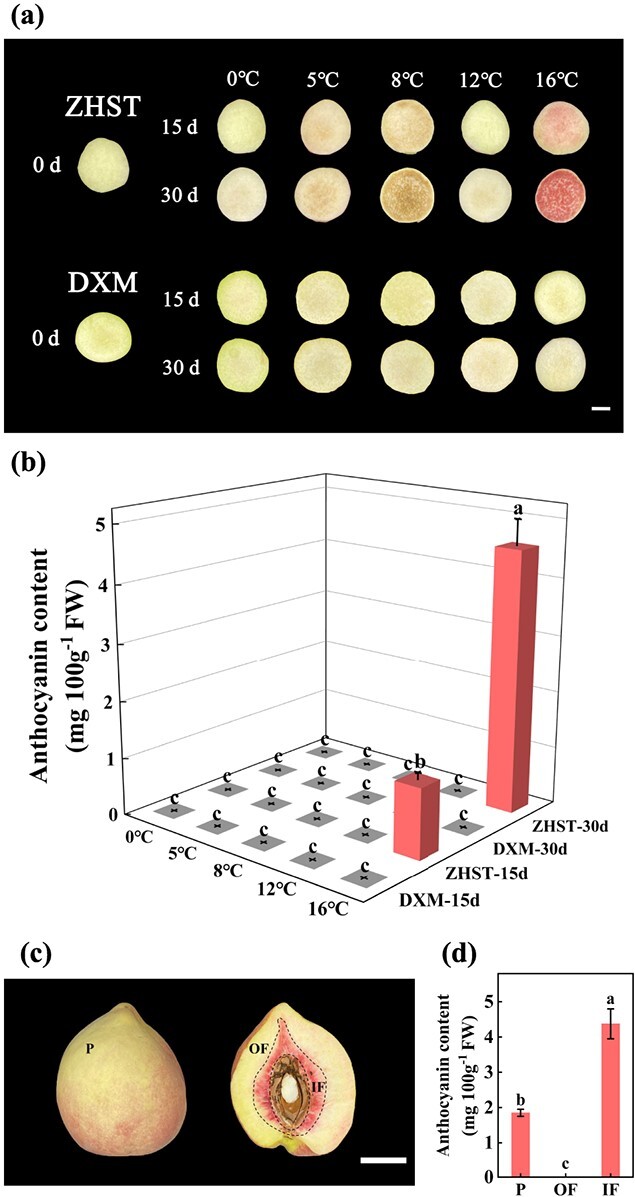
Appearance and accumulation of anthocyanin in fruits of peach cultivars ‘Zhonghuashoutao’ (‘ZHST’) and ‘Dongxuemi’ (‘DXM’) stored at 0, 5, 8, 12, and 16°C for 0, 15, and 30 days. **(a)** Photographs of the outer flesh near the peel (OF). Bar, 2 cm. **(b)** Anthocyanin content in the OF. **(c)** Photographs of three types of fruit tissue, i.e., peel (P), OF and inner flesh around the stone (IF), of ‘ZHST’ at 0 d. Bar, 2 cm. **(d)** Anthocyanin content in different fruit tissues of ‘ZHST’. The experiments were conducted for three times independently, and since the results from different times were similar, the data presented were from one representative experiment. Mean ± SE values were calculated for the data obtained from three independent biological replicates. One-way analysis of variance (ANOVA) testing was performed and different lowercase letters were used to represent statistically significant difference (*P* < 0.05). FW, fresh weight.

Fruits of two peach [*P. persica* (L.) Batsch] cultivars, ‘Zhonghuashoutao’ (‘ZHST’) and ‘Dongxuemi’ (‘DXM’), were stored at different temperatures ranging from 0°C to 16°C. It was found that the OF of ‘ZHST’ stored at 16°C turned slight red at 15  d in storage and deep red at 30 d, but not for fruit stored at 12°C or below (Fig. 1a). However, the OF of ‘DXM’ did not turn red during 30 days of storage at all storage temperatures (Fig. 1a). The content of anthocyanin in the OF of two peach cultivars was consistent with visual appearances (Fig. 1b). Therefore, the accumulation of anthocyanin is cultivar-contrasted and temperature-dependent.

Differential anthocyanin accumulation was observed in different tissue type as well. In mature ‘ZHST’ fruit just prior to storage (0 d), the P was partially red, the OF was not red, and the IF was deep red (Fig. 1c). Consistent with the visual appearance, anthocyanin highly accumulated in IF, whereas anthocyanin was not detected in OF (Fig. 1d). However, in mature ‘DXM’ fruit, the IF was not red, although showed slight browning, and anthocyanin was not detectable (Fig. S1, see online supplementary material). Therefore, the anthocyanin accumulation in ‘ZHST’ fruit is tissue-specific.

### Identification of key genes related to anthocyanin accumulation in peach OF

To explore novel regulatory TFs upstream of *PpMYB10.1*, transcriptome analysis was performed using RNA sequencing with these anthocyanin differentially accumulated tissues. Data analysis of the biological replicates and among peach samples showed high quality and reproducibility of the transcriptome data (Fig. S2, Table S1 and S2, see online supplementary material). Expression of 12 genes, including all seven anthocyanin biosynthetic genes, the gene encoding the transporter PpGST1, regulatory genes *PpMYB10.1* and *PpbHLH3*, as well as two novel TF genes subsequently identified in this study, were analysed by reverse transcription-quantitative PCR (RT-qPCR) and it was found that the expression levels of these genes determined by RNA-seq and RT-qPCR were significantly positively correlated (Fig. S3, see online supplementary material), which further validated the transcriptome data.

**Figure 2:**
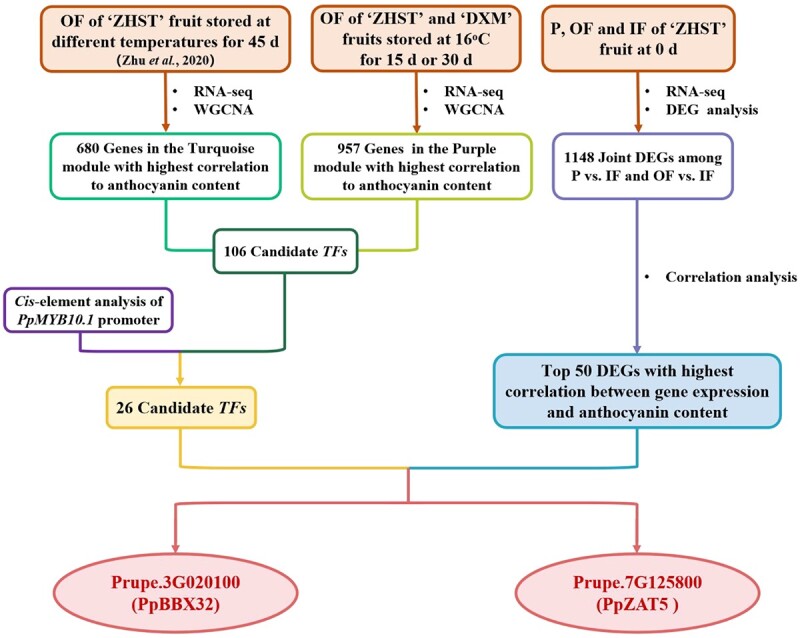
A simplified flowchart presenting the discovery of PpBBX32 and PpZAT5 based on joint analysis of three sets of transcriptome data. DEG, differentially expressed gene; ‘DXM’, ‘Dongxuemi’; IF, inner flesh around the stone; OF, outer flesh near the peel; P, peel; TF, transcription factor; WGCNA, weighted correlation network analysis; ‘ZHST’, ‘Zhonghuashoutao’.

A total of 1496 differentially expressed genes (DEGs) were shared in a comparison between ‘ZHST’ fruit stored at 16°C for 0 d and 30 d, and another comparison between fruit from the two cultivars stored at 16°C for 30 d (Fig. S4a, see online supplementary material). Subsequently, weighted correlation network analysis (WGCNA) was performed using these 1496 DEGs and the modules with high similar expression were combined (Fig. S4b, see online supplementary material). The analysis of module-trait relationships revealed that a collection of 957 genes was included in module Purple (MEpurple) where the gene expression was highest correlated with anthocyanin content (Fig. S4c, see online supplementary material). When this was combined with the previously obtained WGCNA results of transcriptome data on the OF of ‘ZHST’ fruit stored at 0, 5, 8, 12, and 16°C for 45 d [16], as well as the results from the *in silico* prediction of *cis*-acting elements in *PpMYB10.1* promoter, a total of 26 putative upstream transcription factors that may regulate *PpMYB10.1* expression were identified (Fig. 2; Table S3, see online supplementary material).

Expression analysis of anthocyanin biosynthesis-related genes (Fig. S5, see online supplementary material) and DEGs (Fig. S6, see online supplementary material) in the three types of fruit tissue of ‘ZHST’ fruit showed that most genes were expressed differently in the IF as compared to the other two fruit tissue types. Venn diagram analysis of DEGs was further performed and 1148 DEGs related to anthocyanin accumulation in the IF were identified (Fig. S7, see online supplementary material). When this was combined with the correlation analysis results of the anthocyanin content of the P, OF and IF of ‘ZHST’ fruit at harvest, the top 50 genes with highest correlation between expression and anthocyanin content were screened, of which 11 were TFs. Among these, BBX and C2H2 zinc finger were the two most frequently occurring TF families (Fig. 2; Table S4, see online supplementary material). When comparing the list of putative upstream TFs from two independent screenings, only two TFs—Prupe.3G020100 and Prupe.7G125800—were included in both lists (Fig. 2; Tables S3 and S4, see online supplementary material), and based on phylogenetic analysis and sequence alignment information, they were named as PpBBX32 and PpZAT5, respectively (Fig. S8, see online supplementary material).

### Functional characterization of *PpBBX32* and *PpZAT5* in peach fruit and tobacco

**Figure 3:**
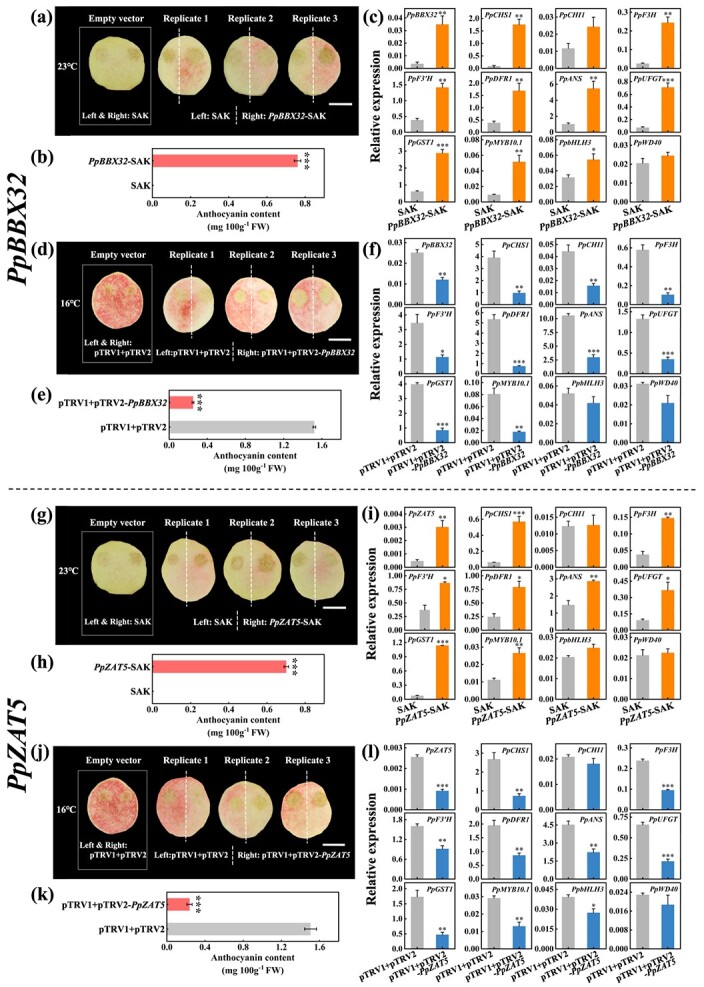
Functional characterization of *PpBBX32* and *PpZAT5* in peach fruit. **(a)**, **(d)**, **(g)**, **(j)** Fruit phenotype and **(b)**, **(e)**, **(h)**, **(k)** anthocyanin content of transient over-expression of *PpBBX32*  **(a)**, **(b)**, *PpZAT5*  **(d)**, **(e)** and virus-induced gene silencing (VIGS) of *PpBBX32*  **(g)**, **(h)**, *PpZAT5*  **(j)**, **(k)** in the outer flesh near the peel (OF) of ‘Zhonghuashoutao’ (‘ZHST’) fruit. **(c)**, **(f)**, **(i)**, **(l)** Relative expression levels of anthocyanin-related genes in the flesh tissues around the injection sites. Peach fruits for transient over-expression assay were stored at 23°C, while fruits for VIGS assay were stored at 16°C. The photograph was taken one week after injection. Bar, 2 cm. The experiments were conducted for three times independently, and since the results from different times were similar, the data presented were from one representative experiment. Mean ± SE values were calculated for the data obtained from three independent biological replicates. Unpaired two-sample Student’s *t*-test was performed and significant difference (*P < 0.05; **P < 0.01; and ***P < 0.001) was indicated with asterisks. FW, fresh weight.

Because a stable genetic transformation system is not available for peach, transient analysis as well as tobacco stable transformation were applied to verify the function of *PpBBX32* and *PpZAT5* in the regulation of anthocyanin accumulation. Through transient over-expression in ‘ZHST’ fruit, we found that *PpBBX32* can significantly promote anthocyanin accumulation (Fig. 3a and b), accompanied by a significant increase in the expression of anthocyanin-related genes (Fig. 3c). As expected, down-regulation of *PpBBX32* via virus-induced gene silencing (VIGS) reduced anthocyanin accumulation, as well as the expression of anthocyanin-related genes (Fig. 3d–f). Similarly, we found that transient over-expression of *PpZAT5* promoted anthocyanin accumulation while VIGS produced an opposite effect (Fig. 3g, h, j, k), and the expression of related genes were consistent with anthocyanin content (Fig. 3i and l). These results indicated that both *PpBBX32* and *PpZAT5* could promote anthocyanin accumulation in ‘ZHST’ peach OF.

**Figure 4:**
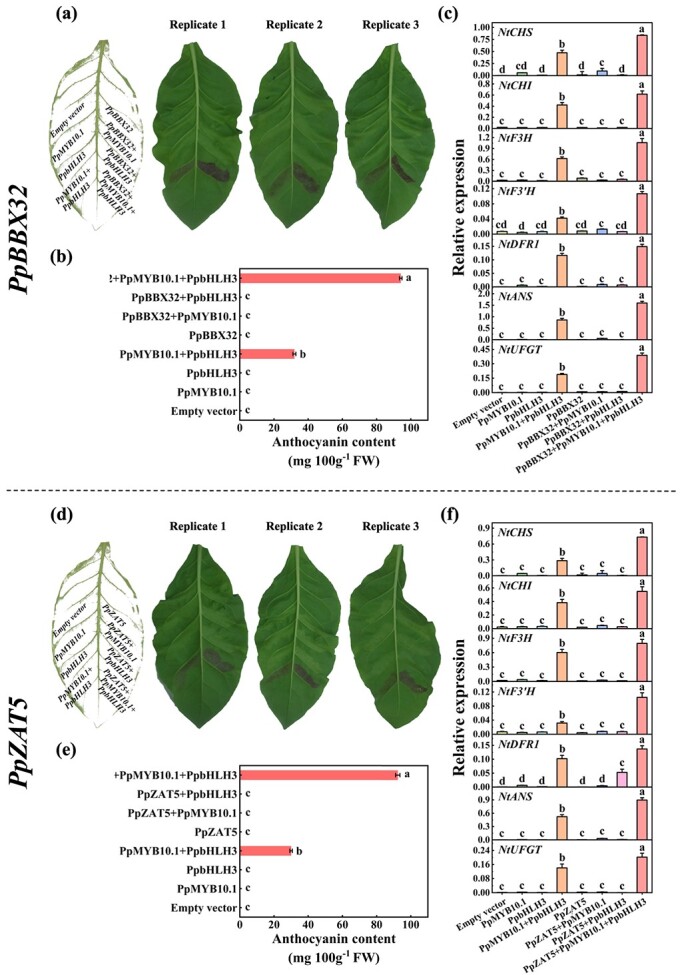
Functional characterization of *PpBBX32* and *PpZAT5* in tobacco leaf. **(a)**, **(d)** Digital images of tobacco (*Nicotiana tabacum*) leaves taken at five days after infiltration. Three biological replicates were set and three leaves from a same plant served for each replicate. The experiments were independently conducted for three times. Similar results were obtained and data from one experiment were presented. **(b)**, **(e)** Total anthocyanin content of leaves at infiltration sites. **(c)**, **(f)** Relative expression of anthocyanin related genes at infiltration sites. Mean ± SE values were calculated for the data obtained from three independent biological replicates. One-way analysis of variance (ANOVA) testing was performed and different lowercase letters were used to represent statistically significant difference (*P* < 0.05). FW, fresh weight.

Transient expression assays were then performed in tobacco leaves. It was found that PpBBX32 and PpZAT5 can enhance the activation effect of PpMYB10.1 plus PpbHLH3 on anthocyanin accumulation (Fig. 4a, b, d, e). The expression of all seven tobacco genes encoding anthocyanin biosynthetic enzymes was higher in areas injected with *PpMYB10.1* plus *PpbHLH3*, and even higher in co-infiltrations further involving either *PpBBX32* or *PpZAT5* (Fig. 4c and f). Therefore, it was demonstrated that PpBBX32 and PpZAT5 have the potential to induce the expression of endogenous pathway genes in tobacco, thereby enhancing the production of anthocyanin.

**Figure 5:**
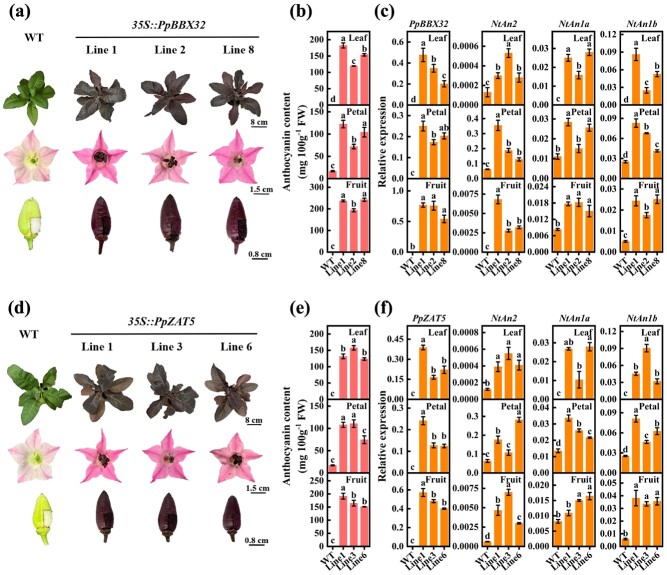
Stable transformation of *PpBBX32* and *PpZAT5* in tobacco (*Nicotiana tabacum*). **(a)**, **(d)** Phenotypes of transgenic tobacco leaf, full-boom flower and fruit (pericarp and seed) overexpressing *PpBBX32*  **(a)** and *PpZAT5*  **(d)***.*  **(b)**, **(e)** Total anthocyanin content of *PpBBX32*  **(b)** and *PpZAT5*  **(e)** in transgenic tobacco leaves, petals and fruits (pericarps and seeds). **(c)**, **(f)** Expression of *PpBBX32*  **(c)**, *PpZAT5*  **(f)**, and tobacco anthocyanin accumulation regulatory genes **(c)**, **(f)** in leaves, flowers, and fruits (pericarps and seeds) of wild-type (WT) and transgenic plants. Mean ± SE values were calculated for the data obtained from three independent biological replicates. One-way analysis of variance (ANOVA) testing was performed and different lowercase letters were used to represent statistically significant difference (*P* < 0.05). FW, fresh weight.

The roles of *PpBBX32* and *PpZAT5* in facilitating anthocyanin accumulation were further confirmed through stable transformation in tobacco. The leaves, flowers, young fruit and seeds of the transgenic lines of *35S::PpBBX32* and *35S::PpZAT5* displayed deep red color, whereas the wild type (WT) plants had green leaves, white seeds, and light red colored petals (Fig. 5a and d). In transgenic lines, the anthocyanin contents of the above-mentioned tissues were higher compared to those in WT (Fig. 5b and e). According to RT-qPCR results, transgenic tobacco plants had a higher transcript level of *PpBBX32*, *PpZAT5*, *NtAn2* (the tobacco anthocyanin MYB regulator), as well as *NtAn1a* and *NtAn1b* (two bHLH regulators involved in tobacco anthocyanin accumulation) as compared with WT (Fig. 5c and f; Fig. S9, see online supplementary material). All these data support the conclusion that *PpBBX32* and *PpZAT5* are positive regulators of anthocyanin accumulation.

### PpBBX32 and PpZAT5 activate *PpMYB10.1* expression via direct binding to its promoter

**Figure 6:**
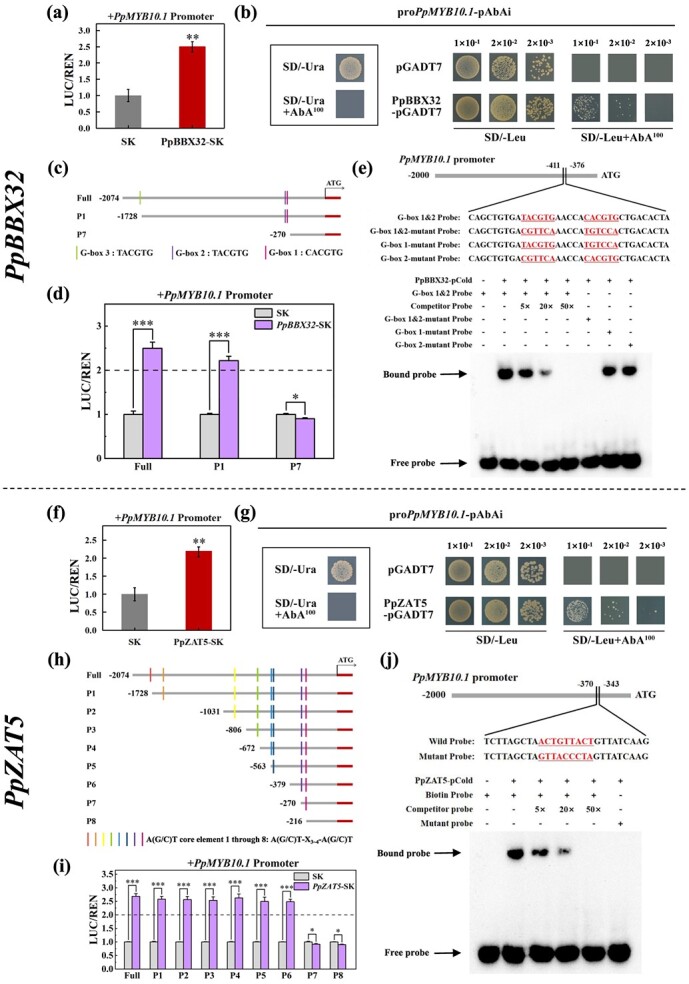
Interaction of PpBBX32 and PpZAT5 with the promoter of *PpMYB10.1*. **(a)**, **(f)** Effects of PpBBX32 **(a)** and PpZAT5 **(f)** on the promoter activity of *PpMYB10.1* measured by dual-luciferase assays. **(b)**, **(g)** Yeast one-hybrid (Y1H) assays on the interactions of PpBBX32 **(b)** and PpZAT5 **(g)** with the promoter of *PpMYB10.1*. **(c)**, **(h)** The predicted G-box motifs **(c)** and A(G/C)T core elements **(h)** indicated with different colored boxes. **(d)**, **(i)** Transactivation activities of PpBBX32 **(d)** and PpZAT5 **(i)** on different truncated *PpMYB10.1* promoters as detected by dual-luciferase assays. **(e)**, **(j)** Electrophoretic mobility shift assay (EMSA) for evaluating the binding of PpBBX32 **(e)** and PpZAT5 **(j)** to the promoter of *PpMYB10.1*. The experiments were conducted for three times independently, and since the results from different times were similar, the data presented were from one representative experiment. Mean ± SE values were calculated for the data obtained from three independent biological replicates. Unpaired two-sample Student’s *t*-test was performed and significant difference (*P < 0.05; **P < 0.01; and ***P < 0.001) was indicated with asterisks. AbA, aureobasidin A; REN, renilla; SD, synthetic dextrose.

After clarifying the function of *PpBBX32* and *PpZAT5*, we explored their regulatory mechanisms by conducting dual-luciferase assays. The activity of *PpMYB10.1* promoter was induced by PpBBX32 or PpZAT5, by 1.5-and 1.2-fold, respectively (Fig. 6a and f). The effects on anthocyanin-related structural genes were investigated as well and it was found that neither *PpBBX32* nor *PpZAT5* could activate the promoters of *PpDFR1*, *PpANS*, *PpUFGT*, and *PpGST1* (Fig. S10, see online supplementary material). Yeast one-hybrid (Y1H) assay was further conducted to investigate the potential direct binding of PpBBX32 or PpZAT5 to the *PpMYB10.1* promoter. It was observed that both PpBBX32 and PpZAT5 can physically bind to the *PpMYB10.1* promoter (Fig. 6b and g). Furthermore, dual-luciferase and Y1H assays were performed to evaluate whether there is a protein-DNA interaction between PpBBX32 and PpZAT5, and no such interaction was observed between them, reciprocally (Fig. S11, see online supplementary material).

To identify the binding sites of PpBBX32 and PpZAT5, *in silico* analysis of *PpMYB10.1* promoter sequence was performed and three G-box motifs, potential PpBBX32 binding sites, as well as eight A(G/C)T core elements, potential PpZAT5 binding sites, were identified (Fig. 6c and h). We further performed the dual-luciferase assays to investigate the critical binding site(s) with different truncated *PpMYB10.1* promoters. A notable reduction in transcriptional activity was found upon truncation of the *PpMYB10.1* promoter to −270 bp upstream of the initiation codon (Fig. 6d and i). Hence, the region spanning from −270 to −1728 bp upstream of the initiation codon may be pivotal in facilitating PpBBX32 binding and activation of the *PpMYB10.1* promoter (Fig. 6d). Similarly, the sequence between −270 and −379 bp of the *PpMYB10.1* promoter were suggested to be important for binding with PpZAT5 (Fig. 6i). The protein-DNA interactions between PpBBX32 or PpZAT5 and *PpMYB10.1* were further verified through electrophoretic mobility shift assay (EMSA). Recombinant His-PpBBX32 and His-PpZAT5 proteins were produced (Fig. S12, see online supplementary material) and used in this assay. As presented in Fig. 6, the interaction between PpBBX32 and PpZAT5 with the *PpMYB10.1* promoter weakened as the concentration of the cold probe increased, while the mutant probe was unable to bind, suggesting that PpBBX32 and PpZAT5 can bind to G-box 1&2 (CACGTG and TACGTG) and ACT core element 7 (ACTGTTACT), respectively (Fig. 6e and j). Therefore, PpBBX32 and PpZAT5 can directly bind and activate the *PpMYB10.1* promoter to regulate anthocyanin accumulation in peach fruit.

### Interaction between PpBBX32, PpZAT5, and PpMYB10.1 *in vitro* and *in vivo*

**Figure 7:**
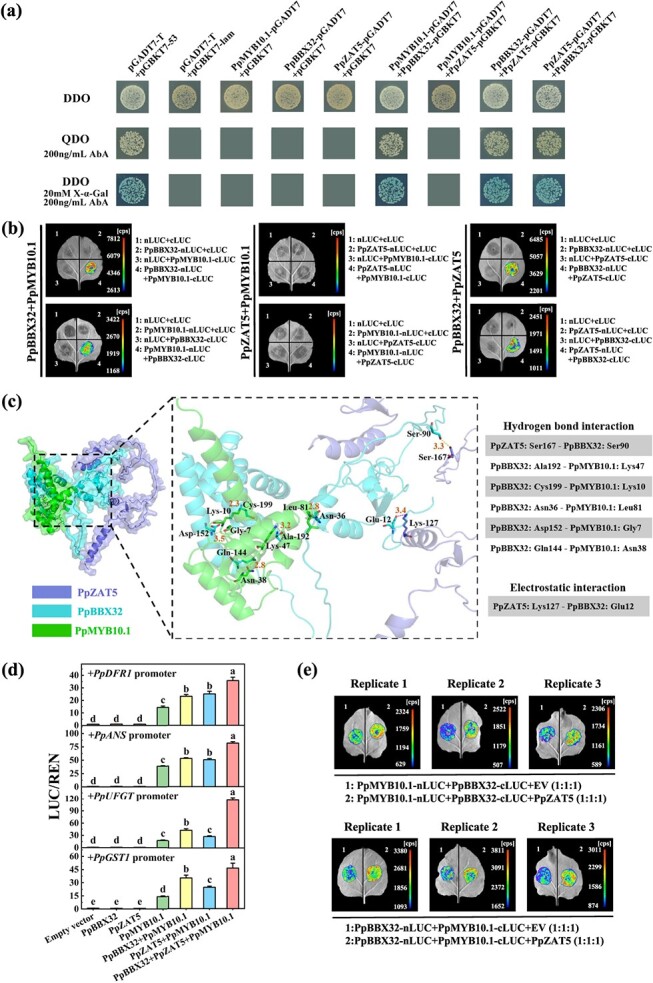
Interaction among PpMYB10.1, PpBBX32 and PpZAT5. **(a)** Yeast two-hybrid (Y2H) assay. **(b)** Firefly luciferase complementation imaging (LCI) in tobacco leaves. **(c)** The predicted protein structures with functional residues demonstrated. Numbers between two amino acids represent bond length in Å. **(d)** Effects of PpMYB10.1, PpBBX32, PpZAT5, alone or in combination, on the promoter activity of genes related to anthocyanin biosynthesis and transport assessed via dual-luciferase assays. **(e)** Effect of the addition of PpZAT5 on the interaction signal between PpBBX32 and PpMYB10.1 measured by LCI assay. Three biological replicates were set and three leaves from a same plant served for each replicate. Mean ± SE values were calculated for the data obtained from three independent biological replicates. One-way analysis of variance (ANOVA) testing was performed and different lowercase letters were used to represent statistically significant difference (*P* < 0.05). AbA, aureobasidin A; cps, count per second; DDO, double dropout; EV, empty vector; QDO, quadruple dropout; REN, renilla; X-α-gal, 5-bromo-4-chloro-3-indolyl-α-D-galactoside.

The protein-DNA interactions between PpBBX32 or PpZAT5 and *PpMYB10.1* were confirmed in the above experiments. To further explore whether PpBBX32, PpZAT5, and PpMYB10.1 can interact at protein level, yeast two-hybrid (Y2H) and luciferase complementation imaging (LCI) assays were performed. Auto-activation was observed for PpMYB10.1-pGBKT7 vector but neither PpBBX32-pGBKT7 nor PpZAT5-pGBKT7 vector on the leucine-deficient synthetic dextrose (SD) medium (SD/−Leu) containing 5-bromo-4-chloro-3-indolyl-α-D-galactoside (X-α-Gal) and 200 ng/mL aureobasidin A (AbA). After transformation with pGADT7 constructs, all co-transformed yeast cells could grow on the SD/−Trp-Leu double dropout (DDO) medium (Fig. 7a). Positive control (pGADT7-T plus pGBKT7–53) and three co-transformants (PpMYB10.1-pGADT7 plus PpBBX32-pGBKT7, PpBBX32-pGADT7 plus PpZAT5-pGBKT7, and PpZAT5-pGADT7 plus PpBBX32-pGBKT7) were able to grow on SD/−Trp-Leu-His-Ade quadruple dropout (QDO) medium containing 200 ng/mL AbA and became blue in the presence of X-α-Gal. However, negative control (pGADT7-T plus pGBKT7-lam) and the other co-transformants were unable to grow on the same selection media (Fig. 7a). This indicates that PpBBX32 physically interacts with PpMYB10.1 and PpZAT5, but PpZAT5 does not interact with PpMYB10.1. The protein interactions were further confirmed through *in vivo* interaction experiments employing LCI. Areas injected with PpBBX32 plus PpMYB10.1 and PpBBX32 plus PpZAT5 showed high fluorescence intensity (Fig. 7b). Data from both assays supported that both PpMYB10.1 and PpZAT5 interacted with PpBBX32 *in vivo*.

Protein–protein interactions were predicted with structures of PpMYB10.1, PpBBX32, and PpZAT5 generated by molecular docking analysis. All possibly functional residues were identified and classified according to their interactions. Two sets of interaction sites were predicted for PpZAT5 and PpBBX32, as were five sets of interaction sites for PpBBX32 and PpMYB10.1 (Fig. 7c). We further explored whether the three proteins impact the activation of anthocyanin biosynthetic genes *PpDFR1*, *PpANS*, and *PpUFGT*, along with the transporter gene *PpGST1*, either individually or in combination with each other. As presented in Fig. 7d, PpMYB10.1 alone can induce promoters of the above-mentioned anthocyanin-related genes, by 14 to 38-fold, and the induction fold increased by around 50% to 23 to 53-fold when PpBBX32 was coupled with PpMYB10.1. PpZAT5 could not increase the activation effect of PpMYB10.1 further, but could significantly enhance the activation effect of PpBBX32 plus PpMYB10.1 on anthocyanin-related genes, by around 50–100% to 35 to 117-fold (Fig. 7d). Furthermore, an enhanced interaction signal from LCI assay was observed when PpZAT5 was further added to the interaction assay between PpBBX32 and PpMYB10.1 (Fig. 7e). All these results suggest that the three proteins form a protein complex in the order PpZAT5-PpBBX32-PpMYB10.1 to promote anthocyanin biosynthesis in peach fruit.

### Exploration of hormonal stimuli regulating PpBBX32 and PpZAT5

**Figure 8:**
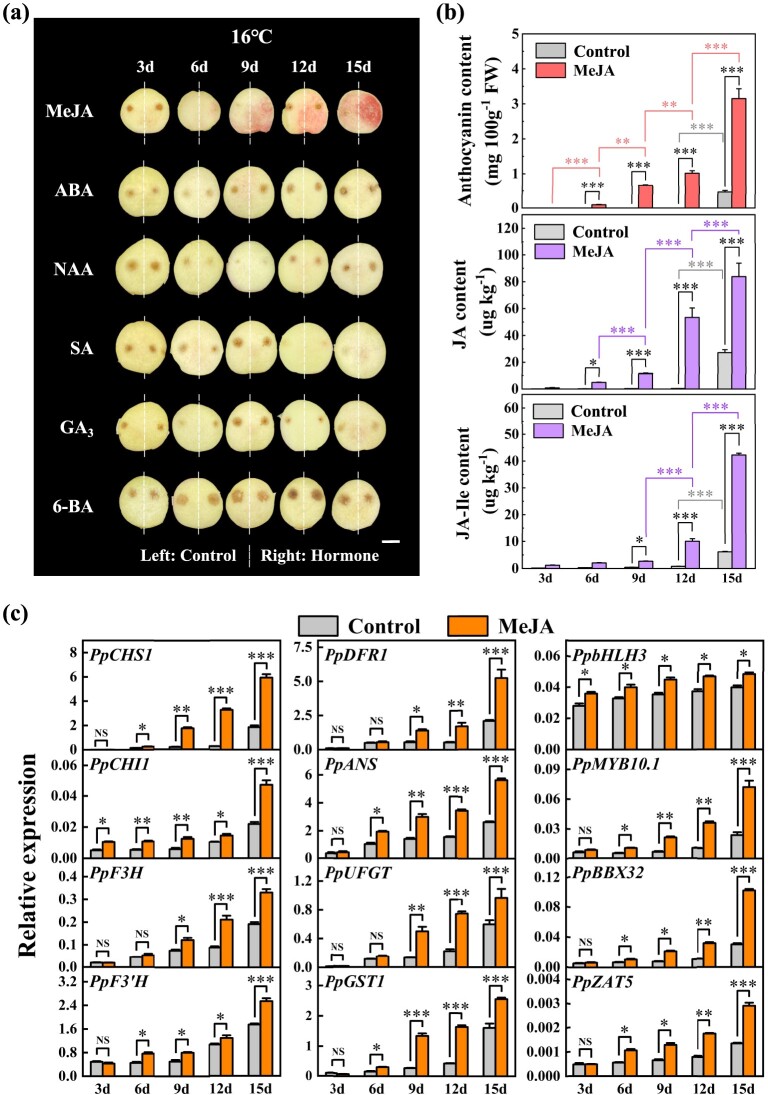
The effects of six plant hormones on induction of anthocyanin in the outer flesh near the peel (OF) of ‘Zhonghuashoutao’ (‘ZHST’) peach at 3–15 d following the infiltration. **(a)** Photographs. Bar, 2 cm. Peach fruits were stored at 16°C. The experiments were conducted for three times independently, and since the results from different times were similar, the data presented were from one representative experiment. **(b)** The contents of anthocyanin, jasmonate (JA) and JA-Ile. **(c)** Relative expression of anthocyanin related genes as well as *PpBBX32* and *PpZAT5*. Mean ± SE values were calculated for the data obtained from three independent biological replicates. Unpaired two-sample Student’s *t*-test was performed and asterisks were used to represent statistically significant difference (*P < 0.05; **P < 0.01; and ***P < 0.001). FW, fresh weight; MeJA, methyl jasmonate; NAA, naphthylacetic acid; SA, salicylic Acid; 6-BA, 6-benzyladenine.

To explore possible upstream endogenous stimuli regulating *PpBBX32* and *PpZAT5* expression, we analyzed the *cis*-acting elements in their promoters. Several elements linked to plant hormone responses were identified (Fig. S13, see online supplementary material), especially ABA and MeJA (Table S5, see online supplementary material). The effects of six common plant hormones—ABA, MeJA, α-naphthalene acetic acid (NAA), salicylic acid (SA), GA_3_, 6-benzyladenine (6-BA)—on induction of anthocyanin in ‘ZHST’ peach OF were tested. As a result, a stimulative effect was observed only from MeJA application (Fig. 8a). The contents of anthocyanin, JA, and JA-Ile in MeJA-infiltrated tissue were significantly increased (Fig. 8b), accompanied with enhanced transcript levels of *PpBBX32*, *PpZAT5*, and anthocyanin-related genes (Fig. 8c). Therefore, JAs possibly function as upstream endogenous stimuli to induce the expression of *PpBBX32* and *PpZAT5* and then downstream genes related to anthocyanin accumulation.

**Figure 9:**
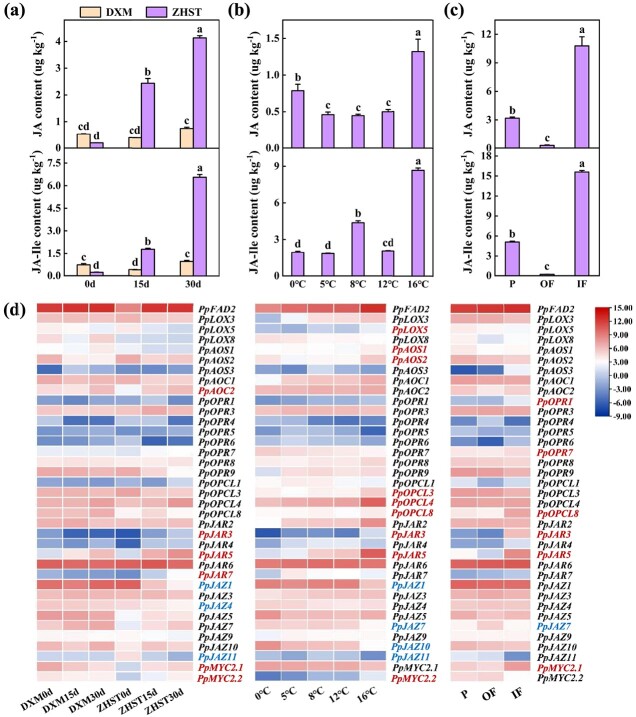
Contents of endogenous jasmonate (JA) and jasmonoyl-isoleucine (JA-Ile) as well as expression of JA biosynthesis and signalling genes in different peach fruits. **(a)**, **(b)**, **(c)** The contents of endogenous JA and JA-Ile in outer flesh near the peel (OF) of ‘Zhonghuashoutao’ (‘ZHST’) and ‘Dongxuemi’ (‘DXM’) stored at 16°C for 30 d **(a)**, OF of ‘ZHST’ stored at different temperatures for 45 d **(b)** and different fruit tissues—peel (P), OF, and inner flesh around the stone (IF)—of ‘ZHST’ at 0 d **(c)**. Mean ± SE values were calculated for the data obtained from three independent biological replicates. One-way analysis of variance (ANOVA) testing was performed and different lowercase letters were used to represent statistically significant difference (*P* < 0.05). **(d)** Transcript levels of genes related to JA biosynthesis and signaling. The colour gradient on the right, ranging from blue, through white, to red represents weak, moderate, and strong gene expression (log_2_FPKM). The expression data were retrieved from RNA-seq results. Genes with expression significantly positively correlated with contents of both anthocyanin and endogenous JA as well as JA-Ile were highlighted in red, while those negatively correlated were in blue (*P* < 0.05). AOC, allene oxide cyclase; AOS, allene oxide synthase; FAD, fatty acid desaturases; JAR, JA-Ile synthetase; JAZ, jasmonate-ZIM domain; LOX, lipoxygenase; MYC2, a kind of basic-helix–loop–helix (bHLH) transcription factor; OPCL, 4-coumarate-CoA ligase-like 5; OPR, 12-oxo-phytodienoic acid reductase.

The content of endogenous JA and JA-Ile, active form of JA, were analysed in tissues of ‘ZHST’ and ‘DXM’ fruits stored at 16°C, ‘ZHST’ stored at different temperatures as well as different fruit tissues of ‘ZHST’ mature fruit. It was found that the contents were higher in anthocyanin accumulating tissues including the OF of ‘ZHST’ fruit stored at 16°C for 15 d or 30 d and the IF of ‘ZHST’ at 0 d (Fig. 9a–c). The anthocyanin content positively correlated significantly with both JA content and JA-Ile content (Fig. S14, see online supplementary material). Furthermore, changes in JA and JA-Ile contents were accompanied by significantly altered expression of several JA biosynthesis and signal transduction-related genes (Fig. 9d). The expression of *PpJAR3* and *PpJAR5*, putatively encoding the enzyme JA-Ile synthetase (JAR) catalyzing the last step of JA-Ile biosynthesis, was highly consistent with anthocyanin accumulation by cultivar, temperature, and tissue type. Transcript levels of *PpMYC2.1*, encoding the putative bHLH TF acting as a master player of JA signaling and an activator of downstream genes, and *PpJAZ7*, encoding a putatively JA inducible JAZMONATE ZIM-DOMAIN (JAZ) TF functioning as a transcriptional repressor, positively and negatively correlated, respectively, with contents of both anthocyanin and endogenous JAs among different cultivars and tissue types. Expression of *PpMYC2.2* positively correlated*,* and *PpJAZ1* and *PpJAZ11* negatively, with different anthocyanin and endogenous JA content among cultivars and storage temperatures. In addition, the transcription of several other genes associated with JA biosynthesis was influenced by at least one of the three factors (Fig. 9d). These results support a role of JAs in differential accumulation of anthocyanin as affected by storage temperature, cultivar, as well as fruit tissue type.

## Discussion

Anthocyanin accumulation in plants is transcriptionally regulated by various TFs with R2R3 MYBs as the basic and core regulators, while other TFs exert their effects directly on anthocyanin biosynthetic and transport genes, or via the MBW complex, or both [2, 8]. For example, direct interaction has been observed between HY5 and the promoters of MYBs and structural genes involved in anthocyanin biosynthesis in various plants [12, 13, 27]. Meanwhile, HY5 also form modules, such as BBX-HY5-MYB and HY5-WRKY-MYB, and contribute to the process of light-induced anthocyanin accumulation [19, 28, 29]. However, there are certain light independent anthocyanin pigmentation processes, such as anthocyanin accumulation in the IF of some peach cultivars and in the inner pericarp of some kiwifruit cultivars [26, 30, 31]. Recently, in IF tissue of ‘Jinxiu’, a yellow-fleshed peach cultivar, it was observed that PpHY5 can increase *PpMYB10.1* transcription via its interaction with PpBBX10 [31]. In the present study, the possible role of PpHY5 in anthocyanin accumulation in ‘ZHST’ fruit was examined. However, anthocyanin accumulation was not associated with the expression of *PpHY5* in peach OF. *PpHY5* expression in the OF of fruits stored at 8°C and 12°C for 45 d was about 1.8 times as compared with that stored at 16°C (Fig. S15a, see online supplementary material), but anthocyanin accumulated only in OF of fruit subjected to 16°C storage [16]. Therefore, the TFs acting upstream of PpMYB10.1 in peach fruit may differ among cultivars and fruit tissue types. Here we reported two novel TFs, PpBBX32 and PpZAT5, as upstream activators of *PpMYB10.1* in flesh, OF and IF, of ‘ZHST’ peach.

BBXs belong to the zinc finger protein superfamily characterized by one or two conserved B-box motifs at the N-terminus [32]. Participation of BBX in regulating anthocyanin accumulation was first reported in *Arabidopsis* [33] and subsequently in apple, pear, and other plants [32]. Expression of anthocyanin-related BBXs is stimulated by light and hence the majority of BBXs play roles in regulating anthocyanin accumulation in peel. For example, MdCOL4, MdCOL11 (MdBBX33), MdBBX20, and MdBBX22 were discovered to modulate the production of anthocyanin in different cultivars of apple [34–37]. In pear, PpyBBX16 was found to positively regulate anthocyanin accumulation, while PpyBBX18 and PpyBBX21 antagonistically regulate anthocyanin biosynthesis by competing for PpyHY5 in the fruit peel [28]. Anthocyanin related BBXs have also been reported in peach. In the presence of PpBBX4, expression of *PpMYB10.1/2/3* was activated by PpHYH, leading to the accumulation of anthocyanin in the sun-exposed peel [38]; PpBBX10 is involved in anthocyanin biosynthesis in IF tissue of ‘Jinxiu’ through the interaction with PpHY5 [31]. In ‘ZHST’, however, it was found that these two *PpBBXs* were not involved in temperature-dependent anthocyanin accumulation in flesh, as the expression was highest for fruit stored at 0°C for 45 d as compared with the other temperatures (Fig. S15b and c, see online supplementary material) while the anthocyanin accumulation was observed only for fruit stored at 16°C [16]. Instead, here we found another BBX member, PpBBX32, took the role of regulating anthocyanin accumulation in ‘ZHST’ flesh. PpBBX32 differs from PpBBX4 and PpBBX10 as well as anthocyanin-related BBXs from other plants as PpBBX32 contains one B-box domain and the others contains two (Fig. S8b, see online supplementary material). PpBBX32 showed high sequence similarity with AtBBX32 and MdBBX37, another two single B-box domain containing BBXs (Fig. S8a and b, see online supplementary material). AtBBX32 acts as a suppressor of photomorphogenesis, by interfering with the protein interaction of BBX21 with HY5, thus suppressing the activity of HY5 and reducing the anthocyanin accumulation [39], and MdBBX37 functions as a repressor of anthocyanin accumulation through its interaction with MdMYB1 and MdMYB9, resulting in a reduction of their binding affinities to the promoters of *MdDFR*, *MdUF3GT*, and *MdANS* [40]. However, PpBBX32 functions as a positive regulator of anthocyanin accumulation by directly binding to the *PpMYB10.1* promoter. The in-depth mechanisms behind such species-specific differences are worthy of further investigation.

ZATs belong to cysteine2/histidine2 (C2H2) zinc finger protein family, with a total of 176 members in *Arabidopsis* [41]. Generally, the C2H2 zinc finger proteins are typically featured with two conserved C2H2 domains, each containing the specific zinc finger motif QALGGH, which is essential for DNA binding [42]. Furthermore, these proteins also possess a conserved ethylene-responsive element-binding factor associated amphiphilic repression (EAR) suppression motif situated at the C-terminus, suggesting their potential role as transcriptional repressors of target genes [43]. C2H2 zinc finger proteins participate in a number of plant metabolism processes, particularly in stress responses and defence activations [41, 43]. Certain specific C2H2 zinc finger proteins, such as *SlZF2* [44], *AtZAT6* [45], and *MdZAT5* [46], were reported to promote anthocyanin accumulation in plants under stress. Interestingly, in pear, *PpyZAT5* has an opposite effect, downregulating *PpyBBX18* expression by binding to the promoter, hence reducing light-induced signal transduction of anthocyanin biosynthesis [47]. However, there has been no report about C2H2 zinc finger proteins regulating anthocyanin accumulation in peach. Here we report that PpZAT5 participates in the regulation of anthocyanin accumulation in ‘ZHST’ flesh. PpZAT5 exhibited high homology and sequence similarity with MdZAT5 and PpyZAT5 (Fig. S8c and d, see online supplementary material). Functional validations and interaction analyses have further demonstrated that *PpZAT5* is a positive regulator (Figs 3–5). Moreover, we further revealed the binding site, A(G/C)T core element (Fig. 6), of PpZAT5 in the *PpMYB10.1* promoter, which adds to the knowledge of the binding site for ZATs, which has been previously reported only in *Arabidopsis* and apple [48, 49].

Besides acting as activators for *PpMYB10.1*, PpBBX32 and PpZAT5 also can interact at protein level, and further with PpMYB10.1 to form the PpZAT5-PpBBX32-PpMYB10.1 protein complex (Fig. 7c), enhancing the anthocyanin accumulation in peach flesh (Fig. 7d and e). This is different from other plants where anthocyanin-related ZATs exert their effects only transcriptionally [45, 47], and BBXs either transcriptionally or via protein interaction but not both [19, 28, 36, 37]. Furthermore, the interaction between a BBX and a MYB has not been reported previously. Taken together, anthocyanin accumulation is cooperatively regulated by multiple TFs via composite mechanisms that vary among plant species.

Plant hormones are one of the main endogenous factors affecting anthocyanin accumulation [10, 11]. A key plant hormone closely related to anthocyanin accumulation is JA. The impact of exogenously applied MeJA on anthocyanin accumulation has been observed in a variety of fruits [11]. In peach, our observations are consistent with the stimulative effect of MeJA on anthocyanin accumulation reported previously [50, 51]. Here we found that JAs may influence the expression of *PpBBX32*, *PpZAT5*, and then other anthocyanin-related genes, thereby promoting anthocyanin biosynthesis in peach flesh (Fig. 8). It has been reported in *Arabidopsis* and apple that JAs facilitated MBW complex formation and therefore anthocyanin accumulation via promoting the release of MYB/MYC subunits as a result of accelerated degradation of JAZs, and repressors of the JA signalling pathway [52, 53]*.* However, TFs, other than MYB and MYC, as targets of JAZ have not been reported. Our study adds to the types of TFs that participated in JA-induced anthocyanin accumulation. In future studies, the function of JA biosynthesis and signal transduction-related genes in peach need to be verified. Moreover, the mechanisms regarding the interaction between PpBBX32/PpZAT5 and JA signal transduction-related genes also deserve further clarification.

Currently, there are numerous studies on the regulation of endogenous JAs content by various internal and external factors [54, 55], as well as reports on the effect of JAs treatment on anthocyanin accumulation [10, 11], but there is a lack of comprehensive research regarding the impact of internal and external factors on anthocyanin accumulation via JA-mediated pathway. Here in peach, we found that internal and external factors such as genotype, temperature, and fruit tissue type may affect endogenous JAs content by affecting the expression of several JA biosynthesis and signal transduction-related genes, eventually leading to differential anthocyanin accumulation in peach flesh (Fig. 9). Further exploration of the specific regulatory mechanisms is underway.

**Figure 10:**
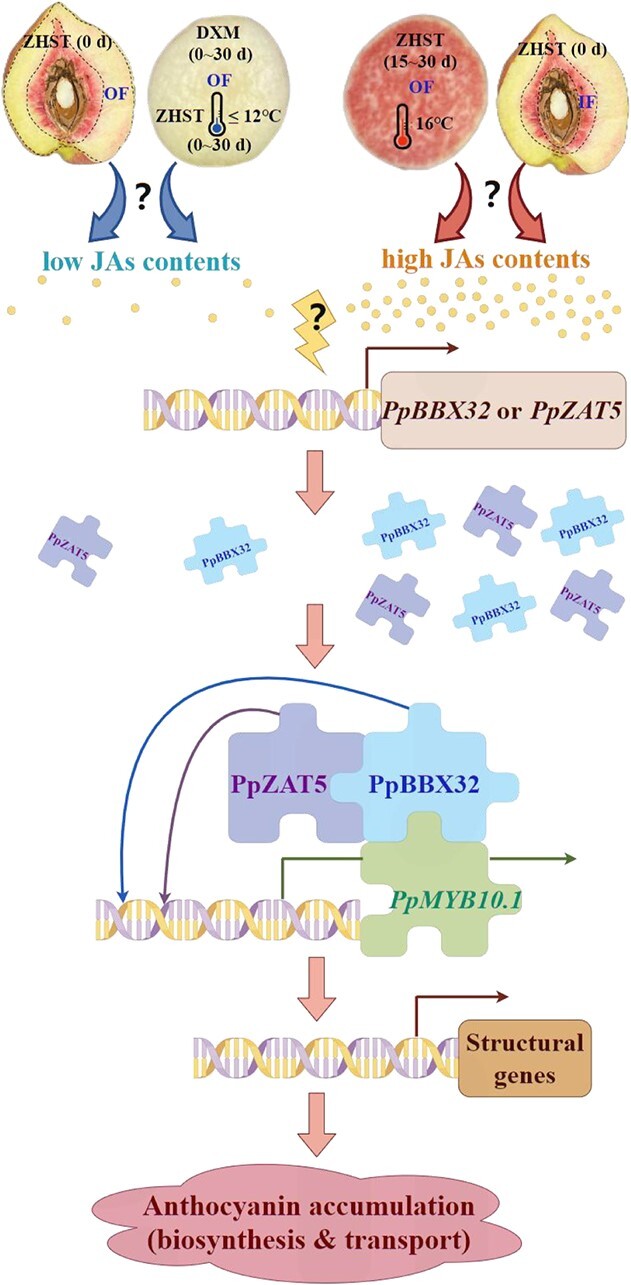
The proposed mechanisms for jasmonates (JAs) inducible PpZAT5 and PpBBX32 modulating cultivar/temperature/tissue-dependent anthocyanin accumulation in peach fruit. The JA content in outer flesh near the peel (OF) of ‘Zhonghuashoutao’ (‘ZHST’) stored at 16°C for 15 d or 30 d and the inner flesh around the stone (IF) of ‘ZHST’ was high, while those in OF of ‘Dongxuemi’ (‘DXM’) stored at a temperature between 0°C and 16°C as well as in OF of ‘ZHST’ stored at ≤12°C was low. The high JAs level stimulated the expression of *PpBBX32* and *PpZAT5*, with detailed mechanisms currently not revealed, and these two TFs then upregulated the expression of *PpMYB10.1* by directly bound to its promoter. Moreover, these two TFs also fulfil their function by forming a protein complex in the order PpZAT5-PpBBX32-PpMYB10.1 which ultimately promoted anthocyanin accumulation in peach.

In conclusion, the OF of ‘DXM’ fruit (stored at a temperature between 0°C and 16°C) and ‘ZHST’ fruit (stored at temperatures ≤12°C, and at maturity stage) have low endogenous JA content; while the OF of ‘ZHST’ fruit (stored at 16°C for 15 d or 30 d) and the IF of mature ‘ZHST’ fruit have high endogenous JAs content. The high content of JAs promotes the expression of *PpBBX32* and *PpZAT5*, both of which function upstream of *PpMYB10.1* and also by forming the PpZAT5-PpBBX32-PpMYB10.1 protein complex to stimulate the expressions of downstream anthocyanin-related genes, and ultimately promote anthocyanin biosynthesis in peach flesh (Fig. 10). This study provides new insights into the mechanisms underlying temperature-dependent and tissue-specific anthocyanin accumulation in peach fruit.

## Materials and methods

### Plant materials and treatment

Mature fruits of two peach [*P. persica* (L.) Batsch] cultivars ‘Zhonghuashoutao’ (‘ZHST’) and ‘Dongxuemi’ (‘DXM’) were picked from an orchard in Zibo (Shandong, China). The fruits were transferred to the lab within 24 h following harvest and stored at 0°C, 5°C, 8°C, 12°C, and 16°C for different days and the OF was sampled. Two batches of ‘ZHST’ fruit were used and the fruits were stored for 45 days for the first batch, which had also been applied in our previous study [16], while 15 and 30 days for the second. For ‘ZHST’ from the second batch and ‘DXM’ at 0 d (i.e., just prior to storage) some extra fruits were sampled as three parts—P, OF, and IF—as shown in Fig. 1c and Fig. S1a (see online supplementary material). The ‘ZHST’ fruit used in VIGS assay were also from the second batch and stored at 16°C for 15 d before injection. Plant hormone treatments were conducted also with ‘ZHST’ at 0 d from the second batch. Six hormones—MeJA (500 mg/L), ABA (500 mg/L), NAA (100 mg/L), SA (200 mg/L), GA_3_ (200 mg/L), and 6-BA (100 mg/L)—were infiltrated into the right side OF of each fruit while the corresponding solvent was infiltrated into the left side as a control. For each infiltration site, 300 μL of solution was injected, and then the fruit were stored at 16°C for 3, 6, 9, 12, and 15 days before photographing and sampling. The OF surrounding the infiltration areas were sampled. For each sampling, 15 fruit were randomly allocated into three biological replicates, with each replicate containing five fruit.

### Anthocyanin extraction and HPLC analysis

The procedure for anthocyanin extraction was conducted following a previously published method [24]. High-performance liquid chromatography (HPLC) analysis was performed with a Waters Alliance 2695 system (Waters Corp., Milford, MA, USA), utilizing a reverse-phase C18 column (4.6 × 250 mm, 5 μm; YMC Co., Ltd., Kyoto, Japan). The absorption at 520 nm was recorded and the quantification of anthocyanin was accomplished by comparing with authentic standard C3G.

### RNA extraction and RT-qPCR analysis

Total RNA extraction from peach tissues and RT-qPCR were performed as reported previously [13]. For tobacco tissues, the TRIzol Reagent Kit (Ambion, Hopkinton, MA, USA) was used. *PpTEF2* (JQ732180) and *NtACT* (AJ421411) were chosen as reference genes to normalize gene expression in peach and tobacco, respectively. Primers used were listed in Table S6 (see online supplementary material). Quantifications were conducted in triplicate for each sample, and the relative expression of genes was analysed via the 2^-ΔCt^ method.

### RNA-seq and its data analysis

RNA-Seq was performed by staff of Novogene Bioinformatics Technology Co. Ltd (Beijing, China). DEGs were identified by applying DESeq R package, with the criteria of |log_2_(fold change)| ≥ 1 and false discovery rate (FDR) ≤ 0.05. WGCNA was performed via the topological overlap metric (TOMtype = ‘unsigned’, minModuleSize = 30, mergeCutHeight = 0.35, power = 12). The heatmap was drawn using TBtools [56].

### Phylogenetic analysis and sequence alignment

Protein sequences from various plants (Table S7, see online supplementary material) were aligned with Muscle tools and then phylogenetic tree was created using the Neighbor-Joining (NJ) method in MEGA 7. The iTOL program (https://itol.embl.de/) was used to visualize the phylogenetic tree. Multiple sequence alignments were visualized using GeneDoc software to visualize multiple sequence alignments.

### *Cis*-acting element analysis

PlantCARE (http://bioinformatics.psb.ugent.be/webtools/plantcare/html/), New PLACE (https://www.dna.affrc.go.jp/PLACE/?action=newplace), and PlantPAN (http://plantpan2.itps.ncku.edu.tw/promoter.php) were applied for the prediction of *cis*-acting elements in the *PpMYB10.1* promoter region. The *cis*-acting element visualization was drawn using TBtools [56].

### Transient overexpression in peach fruit and tobacco leaves

Full-length coding sequences (CDSs) of *PpBBX32*, *PpZAT5*, *PpMYB10.1*, and *PpbHLH3* were amplified from total cDNA of ‘ZHST’ fruit, cloned into the pSAK277 plasmid, and introduced into *Agrobacterium* strain EHA105. Primers used are listed in Table S8 (see online supplementary material). The infiltration was performed using suspension culture of *Agrobacterium* strain EHA105, with three biological replicates, each consisting of either three mature ‘ZHST’ fruit or three 4-to 6-week-old leaves of a same tobacco plant. The experiments were conducted in triplicate, with the infiltrated peach fruits and tobacco plant being placed in a growth room set at 23°C, 75% humidity, 16 h /8 h (light/dark) for 7 d and 5 d, respectively, for phenotypic evaluation and sampling.

### TRV-based VIGS in peach fruit

Full-length CDSs of *PpBBX32* and *PpZAT5* were cloned into the pTRV2 plasmid. Primers used are listed in Table S8 (see online supplementary material). All constructs were introduced into *Agrobacterium* strain GV3101. VIGS assay was performed following a previous reported study [7]. Three biological replicates were set, each consisting of three ‘ZHST’ fruit stored at 16°C for 15 d prior to injection. After injection, the fruit continued to be stored at 16°C for one week and were cut for observation, photographing, and sampling. The experiments were independently conducted three times.

### Tobacco transformation

For tobacco transformation, *Agrobacterium* strain EHA105 harbouring the construct either *PpBBX32*-pSAK277 or *PpZAT5*-pSAK277 were used via a leaf disc co-cultivation method. Samples were collected from leaf, petal of full bloom flower and fruit (pericarp and seed) for anthocyanin amount measurement and gene expression quantification. Three tobacco transgenic lines were served as three biological replicates.

### Dual-luciferase assay

The promoters of *PpMYB10.1*^–2074/−1728/−1031/−806/−672/−563/−379/−270/−216^, *PpBBX32*^−2572^, *PpZAT5*^−2101^, *PpDFR1*^−2391^*, PpANS*^−2294^*, PpUFGT*^−2391^, and *PpGST1*^−2192^, amplifying from ‘ZHST’ genomic DNA, were cloned into the pGreenII 0800-LUC plasmid [57]. Meanwhile, full-length CDSs of *PpBBX32* and *PpZAT5* were cloned into the pGreenII 62-SK plasmid. Primers used are listed in Table S8 (see online supplementary material). Every construct was inserted into *Agrobacterium* strain GV3101. Dual-luciferase assay was conducted utilizing the Dual-Luciferase^@^ Reporter Assay System (Promega, Fitchburg, WI, USA). This assay used tobacco leaves that were 4 weeks to 6 weeks old. Three biological replicates were set with each consisting of six leaf discs (6 mm in diameter) from the same plant.

### Y1H assay

For Y1H assay, 1658 bp, 2101 bp, and 1801 bp of *PpBBX32*, *PpZAT5*, and *PpMYB10.1* promoter, respectively, were cloned into the pAbAi vector. Meanwhile, full-length CDSs of *PpBBX32* and *PpZAT5* were cloned into the pGADT7 vector. Primers used were given in Table S8 (see online supplementary material). Y1H assay was conducted with Matchmaker® Gold Yeast One-Hybrid Library Screening System (Clontech, Palo, CA, USA). To assess interaction, the fused yeast strains were selected on SD/−Leu with the antibiotic AbA.

### Recombinant protein purification and EMSA

The full-length CDSs of *PpBBX32* and *PpZAT5* were inserted into the cloning sites of the pColdTF expression vector (TAKARA, Beijing, China). Recombinant plasmids were transformed into *Escherichia coli* Rossetta (DE3) cell. Subsequently, the cells underwent sonication and the fusion proteins were isolated from the supernatants using HisTALON Gravity Columns (TAKARA, Beijing, China). Biotin labeled probes were synthesized and used in EMSA with the LightShift^@^ Chemiluminescent EMSA kit (Thermo Fisher Scientific, Waltham, MA, USA). The subcloning primers and EMSA probes used are listed in Table S8 (see online supplementary material).

### Y2H assay

Full-length CDSs of *PpBBX32, PpZAT5*, and *PpMYB10.1* were cloned into the pGADT7 and that of *PpBBX32* and *PpZAT5* into pGBKT7. Primer information is listed in Table S8 (see online supplementary material). Y2H assay was conducted using the Matchmaker® Gold Yeast Two-Hybrid Library Screening System (Clontech, Palo, CA, USA). The transformed cells were grown on SD/−Leu/−Trp to confirm the presence of transgenes, whereas transformed cells were grown on SD/−Ade/-His/−Leu/−Trp supplemented with 200 ng/mL AbA, with or without 20 mM of X-α-Gal to test protein–protein interactions.

### LCI assay

The full-length CDS of *PpBBX32*, *PpZAT5*, and *PpMYB10.1* were individually cloned into both pCAMBIA1300-nLUC (nLUC) and pCAMBIA1300-cLUC (cLUC) vectors with primers listed in Table S8 (see online supplementary material). The vectors were transformed into *Agrobacterium* strain EHA105 and used for LCI assay following our previous study [9]. The leaves were injected with 0.2 mM of luciferin two days later and fluorescence signals were monitored using a NightSHADE LB 985 system (Berthold, Bad Wildbad, Germany). Three biological replicates were set and three leaves from the same plant served for each replicate. The experiments were independently conducted three times.

### Molecular docking analysis

The structures of PpBBX32, PpZAT5, and PpMYB10.1 were predicted by Alphafold. The water molecules were eliminated and the polar hydrogen atoms added from the proteins by the AutoDockTools-1.5.7 [58], and then protein–protein docking was performed using Docking Web Server (GRAMM) [59]. The predicted protein–protein complex was again optimized by removing water molecules and adding polar hydrogen atoms by the AutoDockTools-1.5.7. Subsequently, the protein–protein interactions were predicted and the interaction image was generated by PyMOL.

### Determination of JA and JA-Ile contents

Determination of JA and JA-Ile contents was conducted in accordance with a previous study with alterations [60]. In brief, 0.2 g of sample was pulverized in liquid nitrogen, and 2.0 mL of ethyl acetate was added, along with 10 ng of internal standard (D6-JA, Q/C/C; D6-JA-Ile, Q/C/C). After vortexing, the mixture was placed in a light-protected 4°C shaker for 12 h. The supernatant after centrifugation was dried under nitrogen flow, and 1.0 mL of ethyl acetate was added for re-suspension. After centrifuging, the supernatant was evaporated, and finally dissolved in 500 μL of methanol/H_2_O (7:3, v/v). Samples were filtered with 0.22 μm organic filter for subsequent liquid chromatography-mass spectrometry/mass spectrometry (LC–MS/MS) (Agilent Technologies, Santa Clara, CA, USA) analysis. JA and JA-Ile contents were quantified by comparing with the corresponding internal standards.

### Statistical analysis

For each experiment, the mean ± standard error was calculated using at least three biological replicates. Data analysis was conducted through IBM SPSS Statistics 28, employing unpaired two-sample Student’s *t*-test and one-way analysis of variance (ANOVA).

## Supplementary Material

Web_Material_uhae212

## Data Availability

The transcriptome data newly generated in this study have been submitted to the Sequence Read Archive (SRA) database at the National Center for Biotechnology Information (NCBI) with accession numbers PRJNA1092796 and PRJNA1093033.

## References

[1] Mol J, Grotewold E, Koes R. How genes paint flowers and seeds. Trends Plant Sci. 1998;3:212–7

[2] Allan AC, Hellens RP, Laing WA. MYB transcription factors that colour our fruit. Trends Plant Sci. 2008;13:99–102 18280199 10.1016/j.tplants.2007.11.012

[3] He J, Giusti MM. Anthocyanins: natural colorants with health-promoting properties. Annu Rev Food Sci Technol. 2010;1:163–87 22129334 10.1146/annurev.food.080708.100754

[4] Holton TA, Cornish EC. Genetics and biochemistry of anthocyanin biosynthesis. Plant Cell. 1995;7:1071–83 12242398 10.1105/tpc.7.7.1071 PMC160913

[5] Boss PK, Davies C, Robinson SP. Analysis of the expression of anthocyanin pathway genes in developing *Vitis vinifera* L. cv Shiraz grape berries and the implications for pathway regulation. Plant Physiol. 1996;111:1059–66 12226348 10.1104/pp.111.4.1059 PMC160981

[6] Tanaka Y, Sasaki N, Ohmiya A. Biosynthesis of plant pigments: anthocyanins, betalains and carotenoids. Plant J. 2008;54:733–49 18476875 10.1111/j.1365-313X.2008.03447.x

[7] Zhao Y, Dong WQ, Zhu YC. et al. *PpGST1*, an anthocyanin-related glutathione S-transferase gene, is essential for fruit coloration in peach. Plant Biotechnol J. 2019;18:1284–95 31693790 10.1111/pbi.13291 PMC7152611

[8] Albert NW, Davies KM, Lewis DH. et al. A conserved network of transcriptional activators and repressors regulates anthocyanin pigmentation in eudicots. Plant Cell. 2014;26:962–80 24642943 10.1105/tpc.113.122069 PMC4001404

[9] Xue L, Liu XF, Wang WL. et al. MYB transcription factors encoded by diversified tandem gene clusters cause varied *Morella rubra* fruit color. Plant Physiol. 2024;195:598–616 38319742 10.1093/plphys/kiae063

[10] Gu KD, Wang CK, Hu DG. et al. How do anthocyanins paint our horticultural products? Sci Hortic. 2019;249:257–62

[11] Zhao Y, Sun JL, Cherono S. et al. Colorful hues: insight into the mechanisms of anthocyanin pigmentation in fruit. Plant Physiol. 2023;192:1718–32 36913247 10.1093/plphys/kiad160 PMC10315290

[12] Tao RY, Bai SL, Ni JB. et al. The blue light signal transduction pathway is involved in anthocyanin accumulation in ‘red Zaosu’ pear. Planta. 2018;248:37–48 29546452 10.1007/s00425-018-2877-y

[13] Zhao Y, Min T, Chen MJ. et al. The photomorphogenic transcription factor PpHY5 regulates anthocyanin accumulation in response to UVA and UVB irradiation. Front Plant Sci. 2020;11:603178 33537042 10.3389/fpls.2020.603178 PMC7847898

[14] Lin-Wang K, Micheletti D, Palmer J. et al. High temperature reduces apple fruit colour via modulation of the anthocyanin regulatory complex. Plant Cell Environ. 2011;34:1176–90 21410713 10.1111/j.1365-3040.2011.02316.x

[15] Butelli E, Licciardello C, Zhang Y. et al. Retrotransposons control fruit-specific, cold-dependent accumulation of anthocyanins in blood oranges. Plant Cell. 2012;24:1242–55 22427337 10.1105/tpc.111.095232 PMC3336134

[16] Zhu YC, Zhang B, Allan AC. et al. DNA demethylation is involved in the regulation of temperature-dependent anthocyanin accumulation in peach. Plant J. 2020;102:965–76 31923329 10.1111/tpj.14680

[17] Qi TC, Song SS, Ren QC. et al. The jasmonate-ZIM-domain proteins interact with the WD-repeat/bHLH/MYB complexes to regulate jasmonate-mediated anthocyanin accumulation and trichome initiation in *Arabidopsis thaliana*. Plant Cell. 2011;23:1795–814 21551388 10.1105/tpc.111.083261 PMC3123955

[18] Ni JB, Zhao Y, Tao RY. et al. Ethylene mediates the branching of the jasmonate-induced flavonoid biosynthesis pathway by suppressing anthocyanin biosynthesis in red Chinese pear fruits. Plant Biotechnol J. 2020;18:1223–40 31675761 10.1111/pbi.13287 PMC7152598

[19] Bai SL, Tao RY, Tang YX. et al. BBX16, a B-box protein, positively regulates light-induced anthocyanin accumulation by activating *MYB10* in red pear. Plant Biotechnol J. 2019;17:1985–97 30963689 10.1111/pbi.13114 PMC6737026

[20] Ni JB, Wang SM, Yu WJ. et al. The ethylene-responsive transcription factor PpERF9 represses PpRAP2.4 and PpMYB114 via histone deacetylation to inhibit anthocyanin biosynthesis in pear. Plant Cell. 2023;35:2271–92 36916511 10.1093/plcell/koad077 PMC10226596

[21] Liu WJ, Mei ZX, Yu L. et al. The ABA-induced NAC transcription factor MdNAC1 interacts with a bZIP-type transcription factor to promote anthocyanin synthesis in red-fleshed apples. Hort Res. 2023;10:uhad049 10.1093/hr/uhad049 PMC10186271 37200839

[22] Cheng J, Wei GC, Zhou H. et al. Unraveling the mechanism underlying the glycosylation and methylation of anthocyanins in peach. Plant Physiol. 2014;166:1044–58 25106821 10.1104/pp.114.246876 PMC4213075

[23] Ravaglia D, Espley RV, Henry-Kirk RA. et al. Transcriptional regulation of flavonoid biosynthesis in nectarine (*Prunus persica*) by a set of R2R3 MYB transcription factors. BMC Plant Biol. 2013;13:68 23617716 10.1186/1471-2229-13-68 PMC3648406

[24] Zhao Y, Dong WQ, Wang K. et al. Differential sensitivity of fruit pigmentation to ultraviolet light between two peach cultivars. Front Plant Sci. 2017;8:1552 28943881 10.3389/fpls.2017.01552 PMC5596067

[25] Zhou H, Lin-Wang K, Wang HL. et al. Molecular genetics of blood-fleshed peach reveals activation of anthocyanin biosynthesis by NAC transcription factors. Plant J. 2015;82:105–21 25688923 10.1111/tpj.12792

[26] Rahim MA, Busatto N, Trainotti L. Regulation of anthocyanin biosynthesis in peach fruits. Planta. 2014;240:913–29 24827911 10.1007/s00425-014-2078-2

[27] An JP, Qu FJ, Yao JF. et al. The bZIP transcription factor MdHY5 regulates anthocyanin accumulation and nitrate assimilation in apple. Hortic Res. 2017;4:17023 28611922 10.1038/hortres.2017.23 PMC5461414

[28] Bai SL, Tao RY, Yin L. et al. Two B-box proteins, PpBBX18 and PpBBX21, antagonistically regulate anthocyanin biosynthesis via competitive association with *Pyrus pyrifolia* ELONGATED HYPOCOTYL 5 in the peel of pear fruit. Plant J. 2019;100:1208–23 31444818 10.1111/tpj.14510

[29] Mao ZL, Jiang HY, Wang S. et al. The MdHY5-MdWRKY41-MdMYB transcription factor cascade regulates the anthocyanin and proanthocyanidin biosynthesis in red-fleshed apple. Plant Sci. 2021;306:110848 33775373 10.1016/j.plantsci.2021.110848

[30] Wang WQ, Moss SMA, Zeng LH. et al. The red flesh of kiwifruit is differentially controlled by specific activation-repression systems. New Phytol. 2022;235:630–45 35348217 10.1111/nph.18122

[31] Zhao L, Zhang YQ. et al. *PpHY5* is involved in anthocyanin coloration in the peach flesh surrounding the stone. Plant J. 2023;114:951–64 36919360 10.1111/tpj.16189

[32] Gangappa SN, Botto JF. The BBX family of plant transcription factors. Trends Plant Sci. 2014;19:460–70 24582145 10.1016/j.tplants.2014.01.010

[33] Datta S, Hettiarachchi GH, Deng XW. et al. *Arabidopsis* CONSTANS-LIKE3 is a positive regulator of red light signaling and root growth. Plant Cell. 2006;18:70–84 16339850 10.1105/tpc.105.038182 PMC1323485

[34] Bai SL, Saito T, Honda C. et al. An apple B-box protein, MdCOL11, is involved in UV-B-and temperature-induced anthocyanin biosynthesis. Planta. 2014;240:1051–62 25074586 10.1007/s00425-014-2129-8

[35] An JP, Wang XF, Zhang XW. et al. MdBBX22 regulates UV-B-induced anthocyanin biosynthesis through regulating the function of MdHY5 and is targeted by MdBT2 for 26S proteasome-mediated degradation. Plant Biotechnol J. 2019;17:2231–3 31222855 10.1111/pbi.13196 PMC6835122

[36] Fang HC, Dong YH, Yue XX. et al. MdCOL4 interaction mediates crosstalk between UV-B and high temperature to control fruit coloration in apple. Plant Cell Physiol. 2019;60:1055–66 30715487 10.1093/pcp/pcz023

[37] Fang HC, Dong YH, Yue XX. et al. The B-box zinc finger protein MdBBX20 integrates anthocyanin accumulation in response to ultraviolet radiation and low temperature. Plant Cell Environ. 2019;42:2090–104 30919454 10.1111/pce.13552

[38] Zhao L, Sun JL, Cai YM. et al. *PpHYH* is responsible for light-induced anthocyanin accumulation in fruit peel of *Prunus persica*. Tree Physiol. 2022;42:1662–77 35220436 10.1093/treephys/tpac025 PMC9366866

[39] Holtan HE, Bandong S, Marion CM. et al. BBX32, an Arabidopsis B-box protein, functions in light signaling by suppressing HY5-regulated gene expression and interacting with STH2/BBX21. Plant Physiol. 2011;156:2109–23 21632973 10.1104/pp.111.177139 PMC3149924

[40] An JP, Wang XF, Espley RV. et al. An apple B-box protein MdBBX37 modulates anthocyanin biosynthesis and hypocotyl elongation synergistically with MdMYBs and MdHY5. Plant Cell Physiol. 2020;61:130–43 31550006 10.1093/pcp/pcz185

[41] Englbrecht CC, Schoof H, Bohm S. Conservation, diversification and expansion of C2H2 zinc finger proteins in the *Arabidopsis thaliana* genome. BMC Genomics. 2004;5:39 15236668 10.1186/1471-2164-5-39 PMC481060

[42] Kubo KI, Sakamoto A, Kobayashi A. et al. Cys2/His2 zinc-finger protein family of petunia: evolution and general mechanism of target-sequence recognition. Nucleic Acids Res. 1998;26:608–15 9421523 10.1093/nar/26.2.608 PMC147284

[43] Ciftci-Yilmaz S, Mittler R. The zinc finger network of plants. Cell Mol Life Sci. 2008;65:1150–60 18193167 10.1007/s00018-007-7473-4 PMC11131624

[44] Hichri I, Muhovski Y, Zizkova E. et al. The *Solanum lycopersicum* zinc Finger2 cysteine-2/histidine-2 repressor-like transcription factor regulates development and tolerance to salinity in tomato and arabidopsis. Plant Physiol. 2014;164:1967–90 24567191 10.1104/pp.113.225920 PMC3982756

[45] Shi H, Liu G, Wei Y. et al. The zinc-finger transcription factor ZAT6 is essential for hydrogen peroxide induction of anthocyanin synthesis in *Arabidopsis*. Plant Mol Biol. 2018;97:165–76 29675814 10.1007/s11103-018-0730-0

[46] Wang DR, Yang K, Wang X. et al. Overexpression of MdZAT5, an C2H2-type zinc finger protein, regulates anthocyanin accumulation and salt stress response in apple calli and *Arabidopsis*. Int J Mol Sci. 2022;23:1897–913 35163816 10.3390/ijms23031897 PMC8836528

[47] Zhang L, Tao RY, Wang SM. et al. PpZAT5 suppresses the expression of a B-box gene *PpBBX18* to inhibit anthocyanin biosynthesis in the fruit peel of red pear. Front Plant Sci. 2022;13:1022034 36304405 10.3389/fpls.2022.1022034 PMC9592862

[48] Sakamoto H, Maruyama K, Sakuma Y. et al. *Arabidopsis* Cys2/His2-type zinc-finger proteins function as transcription repressors under drought, cold, and high-salinity stress conditions. Plant Physiol. 2004;136:2734–46 15333755 10.1104/pp.104.046599 PMC523337

[49] Yu L, Liu XF, Guo ZW. et al. Interaction between MdZAT10a-like and MdbHLH100 negatively regulates salt tolerance in apple (*Malus domestica* Borkh.). Environ Exp Bot. 2022;200:104938

[50] Saniewski M, Miyamoto K, Ueda J. Methyl jasmonate induces gums and stimulates anthocyanin accumulation in peach shoots. J Plant Growth Regul. 1998;17:121–4

[51] Tang TT, Zhou HS, Wang LB. et al. Post-harvest application of methyl jasmonate or prohydrojasmon affects color development and anthocyanins biosynthesis in peach by regulation of sucrose metabolism. Frontiers Nutrition. 2022;9:871467 10.3389/fnut.2022.871467 PMC9037146 35479735

[52] An XH, Tian Y, Chen KQ. et al. *MdMYB9* and *MdMYB11* are involved in the regulation of the JA-induced biosynthesis of anthocyanin and proanthocyanidin in apples. Plant Cell Physiol. 2015;56:650–62 25527830 10.1093/pcp/pcu205

[53] Xie Y, Tan HJ, Ma ZX. et al. DELLA proteins promote anthocyanin biosynthesis via sequestering MYBL2 and JAZ suppressors of the MYB/bHLH/WD40 complex in *Arabidopsis thaliana*. *Mol Plant*. 2016;9:711–21 26854848 10.1016/j.molp.2016.01.014

[54] Wasternack C, Hause B. Jasmonates: biosynthesis, perception, signal transduction and action in plant stress response, growth and development. Ann Bot. 2013;111:1021–58 23558912 10.1093/aob/mct067 PMC3662512

[55] Ruan JJ, Zhou YX, Zhou ML. et al. Jasmonic acid signaling pathway in plants. Int J Mol Sci. 2019;20:2479 31137463 10.3390/ijms20102479 PMC6566436

[56] Chen CJ, Chen H, Zhang Y. et al. TBtools: an integrative toolkit developed for interactive analyses of gig biological data. Mol Plant. 2020;13:1194–202 32585190 10.1016/j.molp.2020.06.009

[57] Hellens RP, Allan AC, Friel EN. et al. Transient expression vectors for functional genomics, quantification of promoter activity and RNA silencing in plants. Plant Methods. 2005;1:13 16359558 10.1186/1746-4811-1-13 PMC1334188

[58] Morris GM, Huey R, Olson AJ. Using AutoDock for ligand-receptor docking. Current Protocols Bioinformatics. 2008;8:8.14 10.1002/0471250953.bi0814s24 19085980

[59] Katchalski-Katzir E, Shariv I, Eisenstein M. et al. Molecular surface recognition: determination of geometric fit between proteins and their ligands by correlation techniques. Proc Natl Acad Sci USA. 1992;89:2195–9 1549581 10.1073/pnas.89.6.2195 PMC48623

[60] Pan XQ, Welti R, Wang XM. Simultaneous quantification of major phytohormones and related compounds in crude plant extracts by liquid chromatography-electrospray tandem mass spectrometry. Phytochemistry. 2008;69:1773–81 18367217 10.1016/j.phytochem.2008.02.008

