# Supplementary material for: PpBBX32 and PpZAT5 modulate temperature-dependent and tissue-specific anthocyanin accumulation in peach fruit
Source: Hortic Res. 2024 Jul 30;11(10):uhae212. doi: 10.1093/hr/uhae212 (PMC11462610; doi:10.1093/hr/uhae212)
Supplement: Web_Material_uhae212 [file web_material_uhae212.zip › Suppl Figures-HR.pdf]

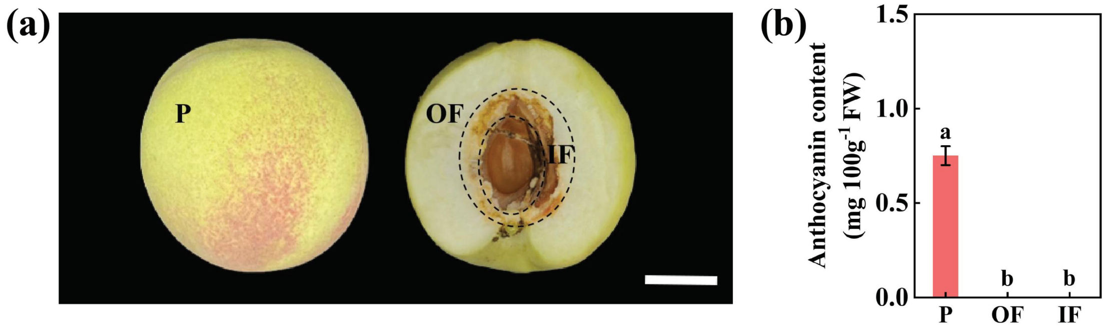

Supporting Information Fig. S1 Appearance and accumulation of anthocyanin in 'DXM' peach fruit at 0 d. (a) Photographs of three types of fruit tissue, i.e., peel (P), the outer flesh near the peel (OF) and inner flesh around the stone (IF). Bar, 2 cm. **(b)** Anthocyanin content in different fruit tissues of 'DXM'. Mean ± SE values were calculated for the data obtained from three independent biological replicates. One-way analysis of variance (ANOVA) testing was performed and different lowercase letters were used to represent statistically significant difference ($P < 0.05$). FW, fresh weight.

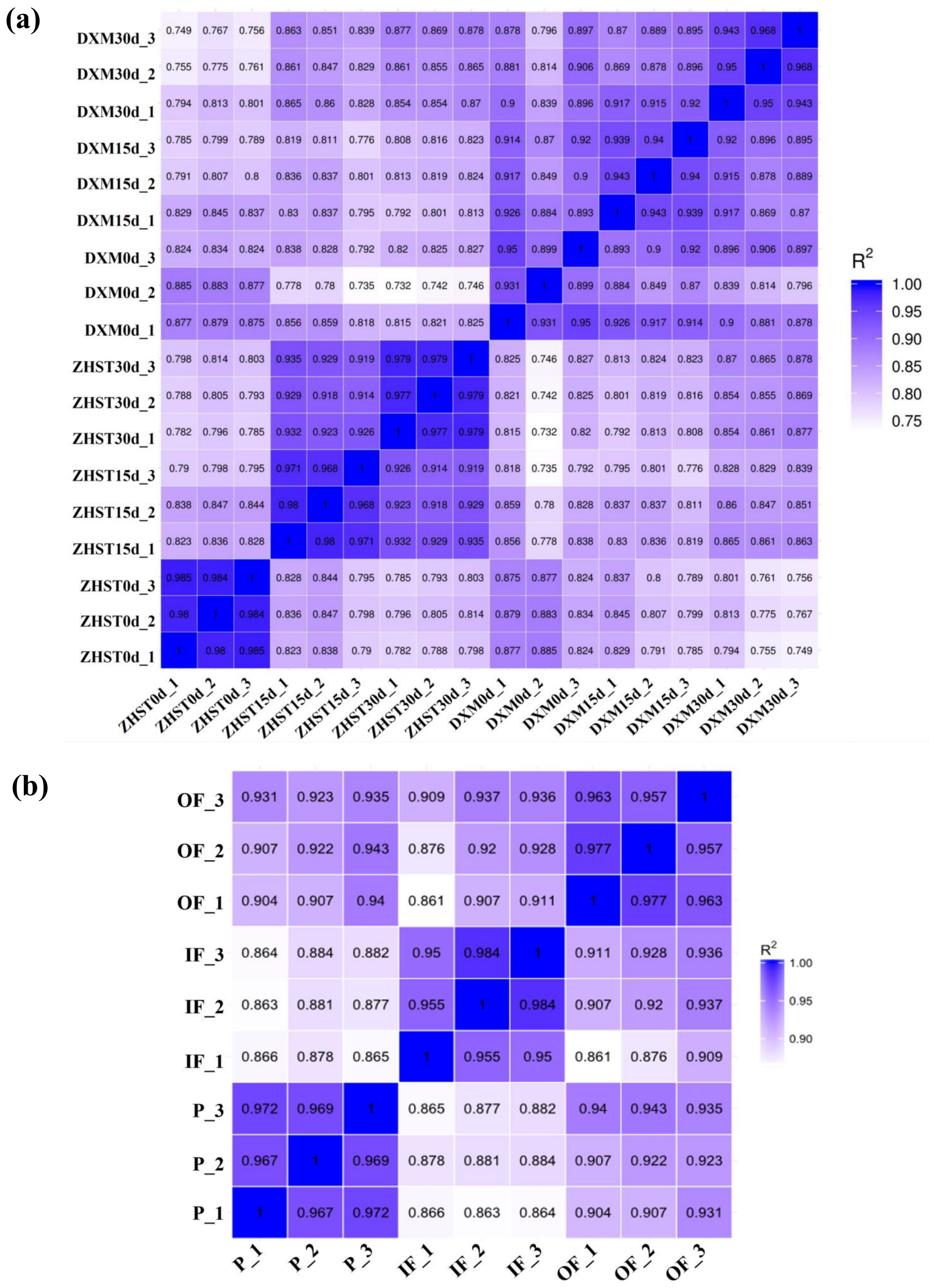

Supporting Information Fig. S2 Correlation matrix between RNA-Seq samples. **(a)** Pearson correlation of transcriptomic data between the outer flesh near the peel (OF) samples of ‘Zhonghuashoutao’ (‘ZHST’) and ‘Dongxuemi’ (‘DXM’) peach stored at 16 ºC for 0, 15 and 30 d. **(b)** Pearson correlation of transcriptomic data between the peel (P), the OF and the inner flesh around the stone (IF) samples of ‘ZHST’ peach fruit at 0 d.

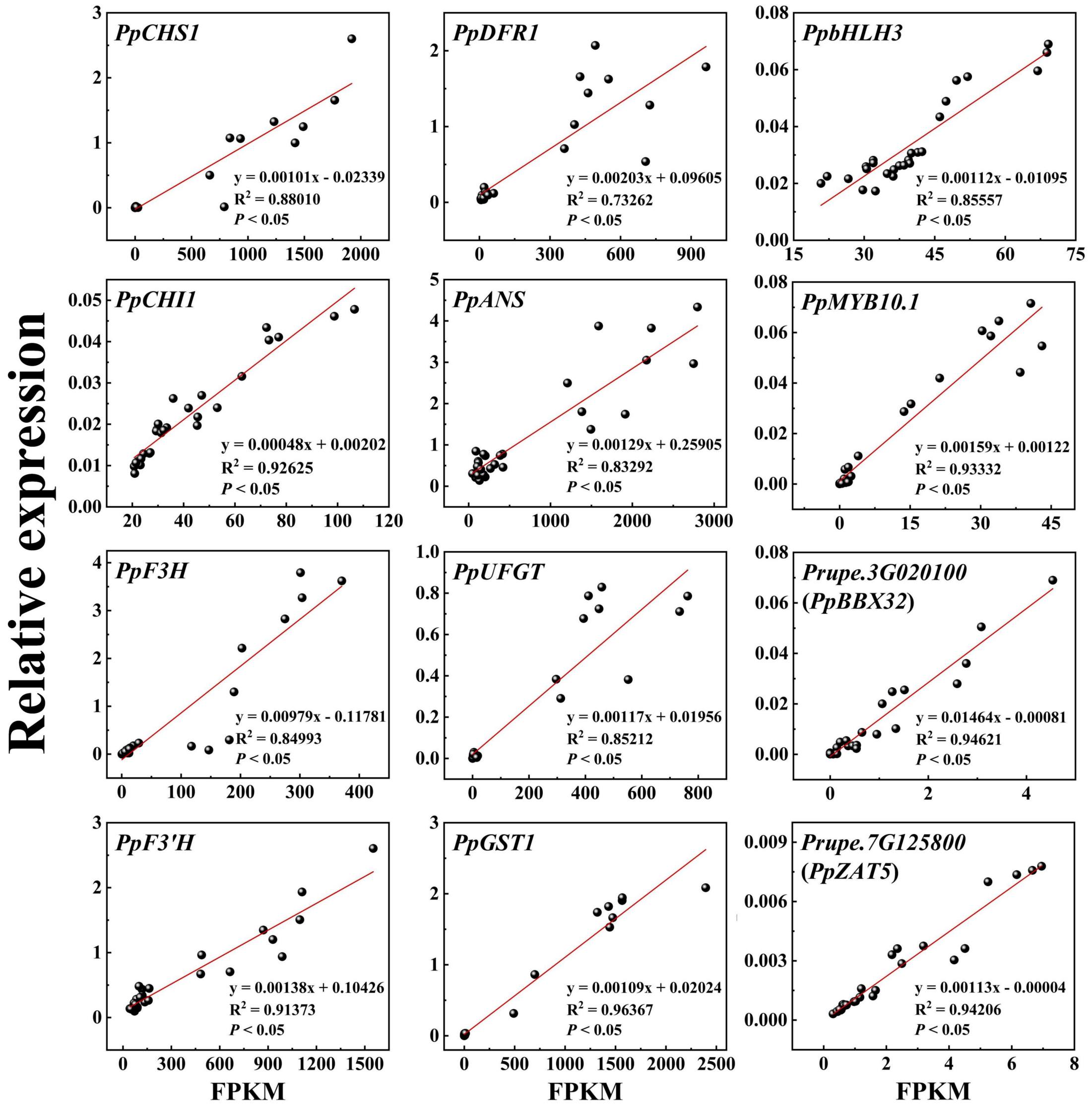

Supporting Information Fig. S3 Expression validation of anthocyanin related genes as well as *PpBBX32* and *PpZAT5* by RT-qPCR. FPKM came from RNA-seq results of the outer flesh near the peel (OF) samples of ‘Zhonghuashoutao’ (‘ZHST’) and ‘Dongxuemi’ (‘DXM’) peach stored at 16 °C for 0, 15 and 30 d, and the peel (P), the OF and the inner flesh around the stone (IF) samples of ‘ZHST’ peach fruit at 0 d. FPKM, expected number of fragments per kilobase of transcript sequence per millions base pairs sequenced.

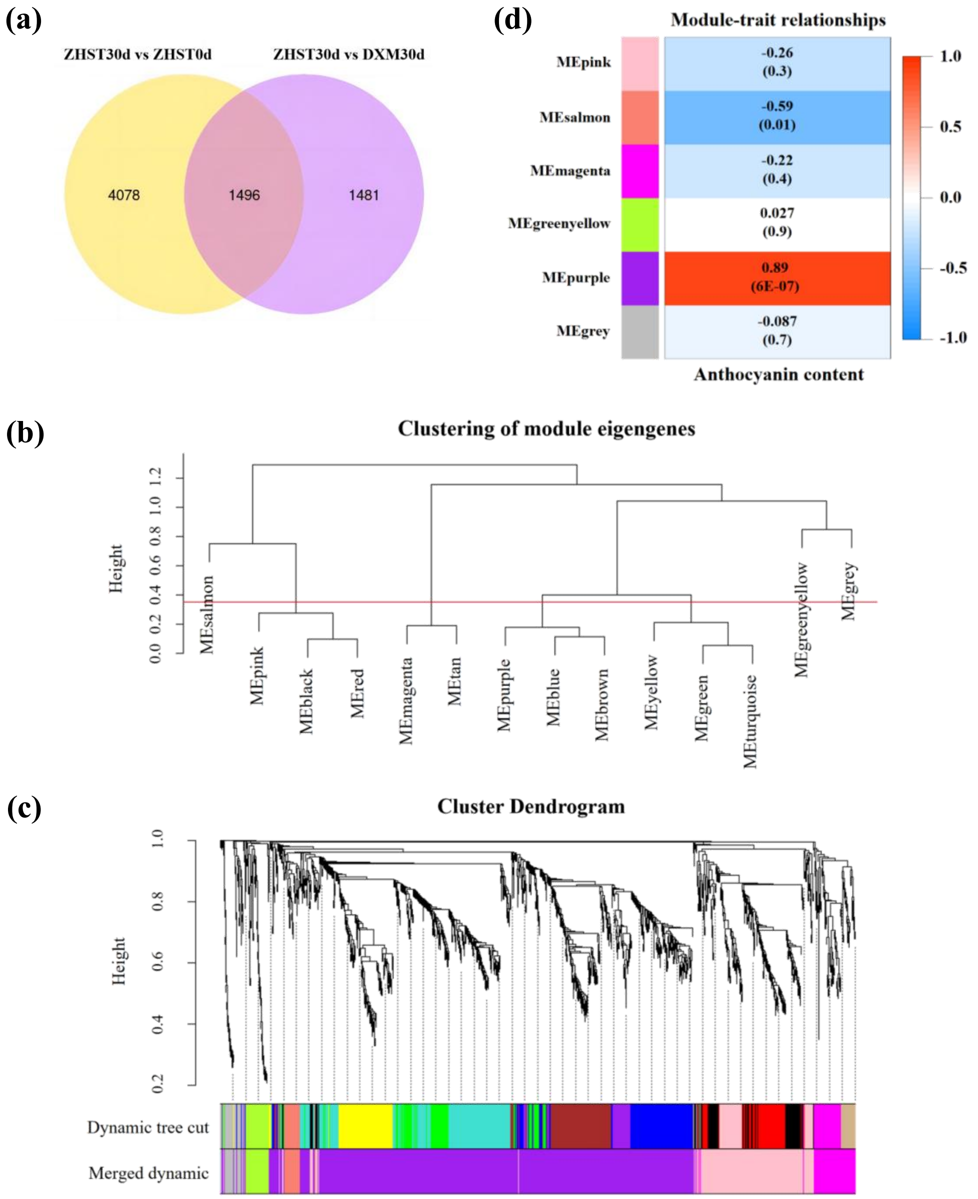

Supporting Information Fig. S4 Bioinformatics analysis of transcriptome data of the two cultivar samples. (a) Venn diagrams of differentially expressed genes (DEGs) revealed by paired comparison the outer flesh near the peel (OF) samples of ‘Zhonghuashoutao’ (‘ZHST’) peach fruits stored at 16 ^{o}C for 0 d and 30 d, as well as the OF samples of ‘ZHST’ and ‘Dongxuemi’ (‘DXM’) peach fruits stored at 16 ^{o}C for 30 d. **(b)** Clustering dendrogram of 1496 DEGs. The red line represents a height cut of 0.35, corresponding to a correlation of 0.65. **(c)** Network analysis dendrogram showing modules identified by weighted gene co-expression network analysis (WGCNA) from 1496 DEGs, including assigned merged module colors and the original module colors. **(d)** Correlation analysis between the module eigengene and anthocyanin content. The left panel shows six modules. The color scale on the right shows module-trait correlation from -1 (blue) to 1 (red).

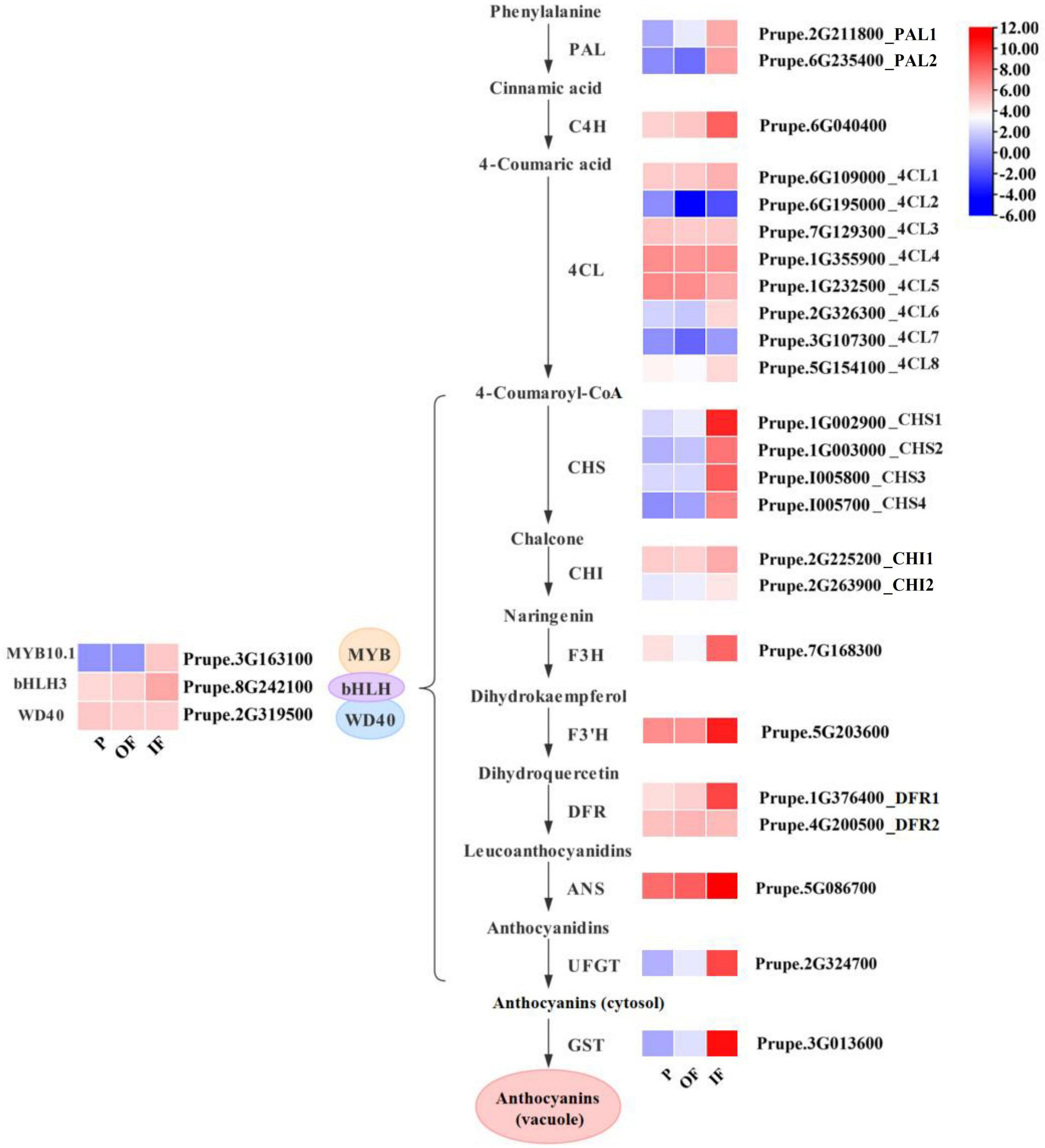

Supporting Information Fig. S5 The expression of anthocyanin biosynthetic and transport genes in the peel (P), the outer flesh near the peel (OF) and the inner flesh around the stone (IF) of 'Zhonghuashoutao' ('ZHST') peach fruit at 0 d. Enzyme name, gene IDs, and expression patterns are indicated on the side of each step for anthocyanin biosynthesis. The colour gradient on the right, ranging from blue, through white, to red represents low, middle, and high values of gene expression (log_2FPKM). FPKM came from RNA-seq results. 4CL, 4-coumarate coenzyme A ligase; ANS, anthocyanidin synthase/leucoanthocyanidin dioxygenase; bHLH, basic helix-loop-helix; C4H, cinnamic acid 4-hydroxylase; CHI, chalcone isomerase; CHS, chalcone synthase; DFR, dihydroflavonol 4-reductase; F3H, flavanone 3-hydroxylase; F3'H, flavanoid 3'-hydroxylase; FPKM, expected number of fragments per kilobase of transcript sequence per millions base pairs sequenced; GST, glutathione *S*-transferase; PAL, phenylalanine ammonia lyase; UFGT, (UDP)-glucose:flavonoid-3-*O*-glycosyltransferase.

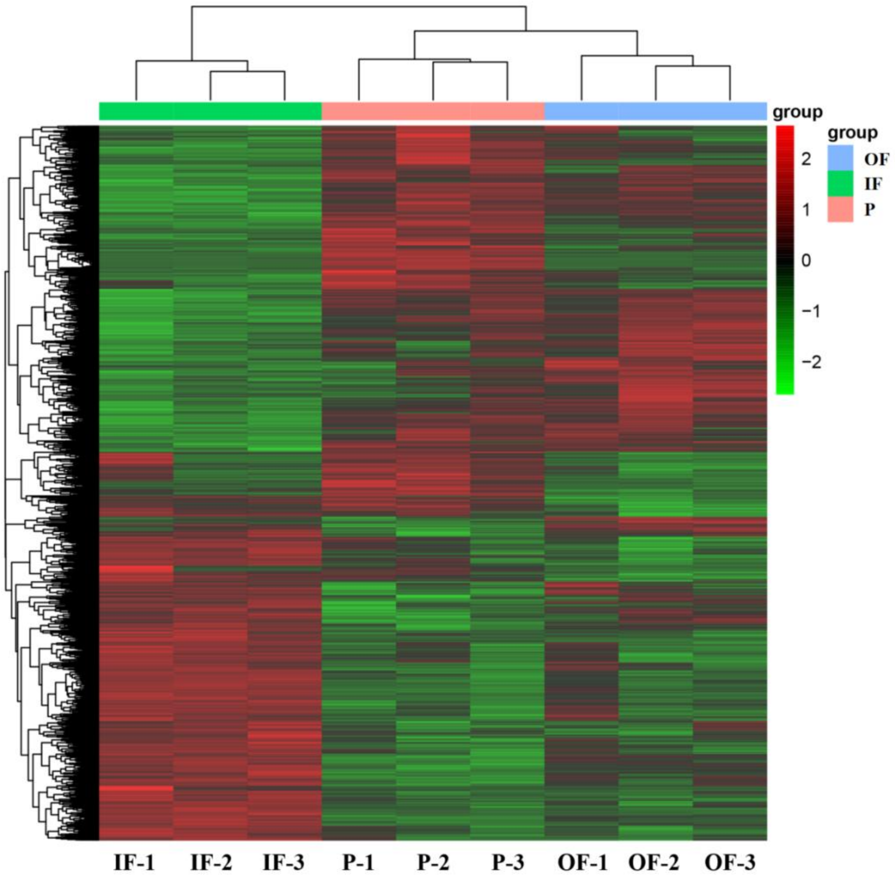

Supporting Information Fig. S6 Hierarchy clustering of differentially expressed genes (DEGs) across three types of fruit tissue of 'Zhonghuashoutao' ('ZHST') peach fruit at 0 d. The rows in the heatmap represent genes, and the columns indicate samples. The colors of heatmap cells indicate scaled expression level of genes (log_2FPKM) across different samples from the peel (P), the outer flesh near the peel (OF) and the inner flesh around the stone (IF). The color gradient, ranging from green, through black, to red represents low, middle and high values of gene expression. FPKM came from RNA-seq results. FPKM, expected number of fragments per kilobase of transcript sequence per millions base pairs sequenced.

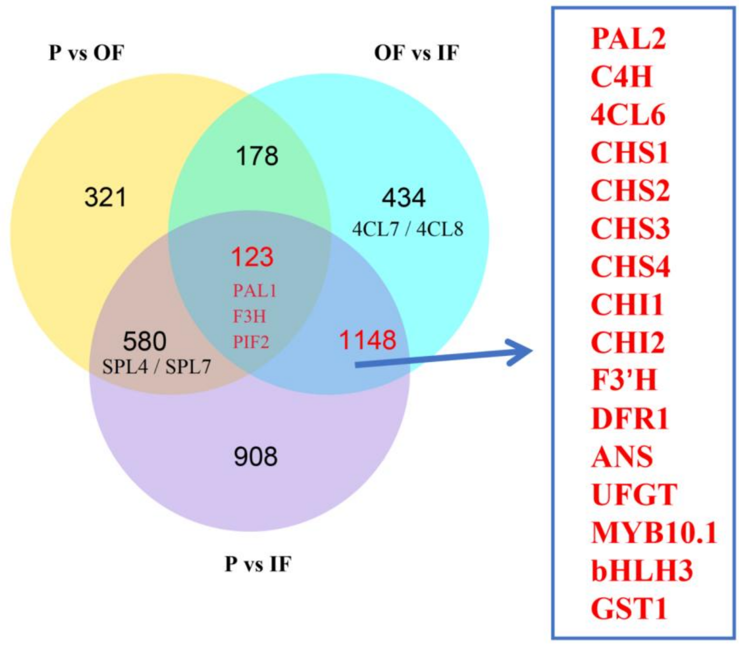

Supporting Information Fig. S7 Venn plots of differentially expressed genes (DEGs) from the comparison of three types of fruit tissue of ‘Zhonghuashoutao’ (‘ZHST’) peach fruit at 0 d. Venn diagrams illustrating the number of DEGs revealed by paired comparison between the peel (P), the outer flesh near the peel (OF) and the inner flesh around the stone (IF). The box on the right shows the anthocyanin biosynthesis-related genes present in 1148 DEGs revealed by paired comparison between P and IF, as well as OF and IF.

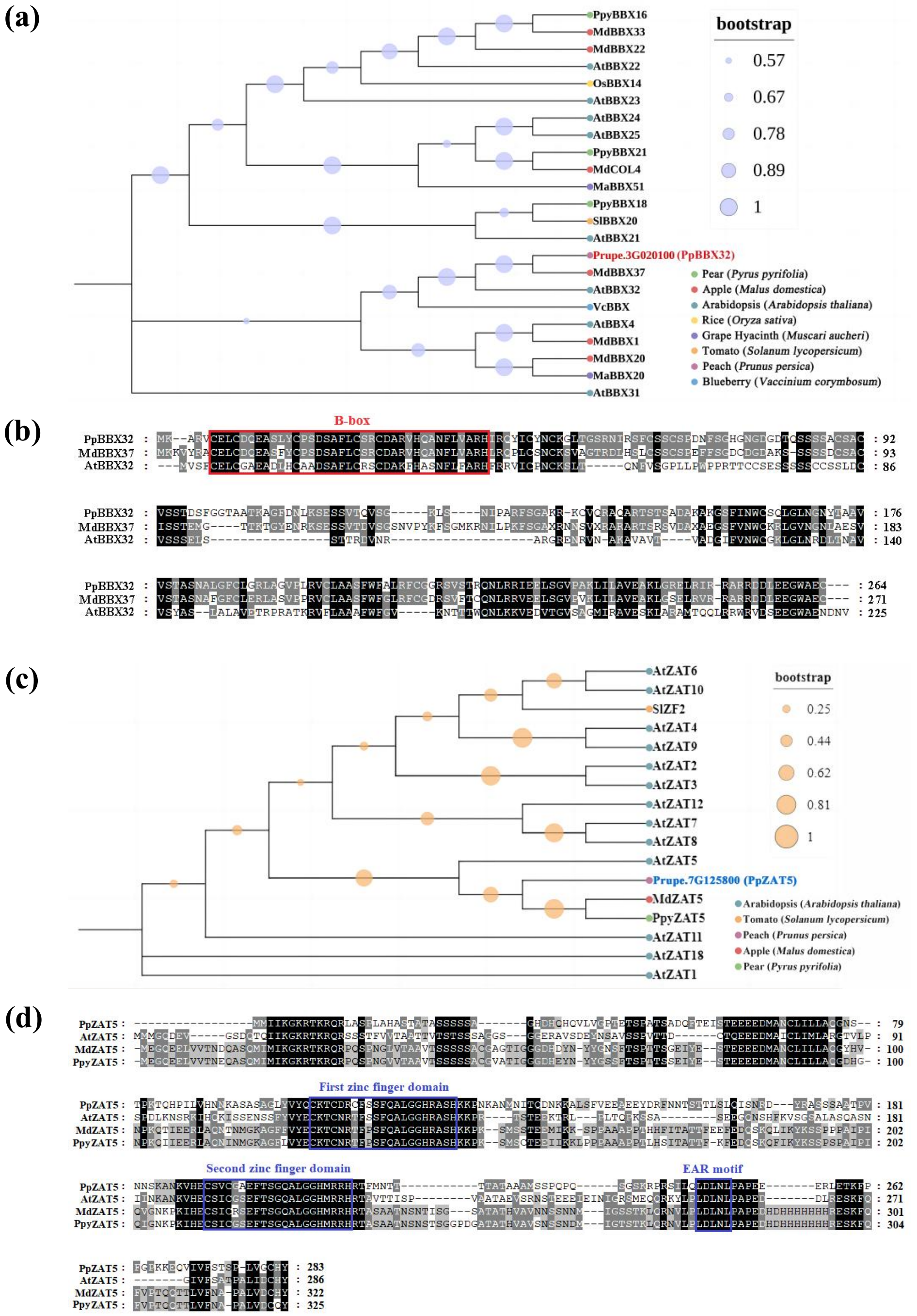

Supporting Information Fig. S8 Phylogenetic and alignment analyses of PpBBX32 and PpZAT5. (a) Phylogenetic tree derived from amino acid sequences of the BBX from peach (Prupe.3G020100) and other plant species reported to be associated with anthocyanin accumulation. **(b)** Alignment of BBXs from peach, apple and Arabidopsis. Red box indicates the conserved B-box domain. **(c)** Phylogenetic analysis of the C2H2-type zinc finger protein from peach (Prupe.7G125800) and other plant species associated with anthocyanin accumulation as well as all Arabidopsis ZATs. **(d)** Alignment of C2H2-type zinc finger proteins from peach, Arabidopsis, apple and pear. Blue boxes display the conserved domains of C2H2-type zinc finger proteins. Accession numbers for the genes involved are given in Table S2.

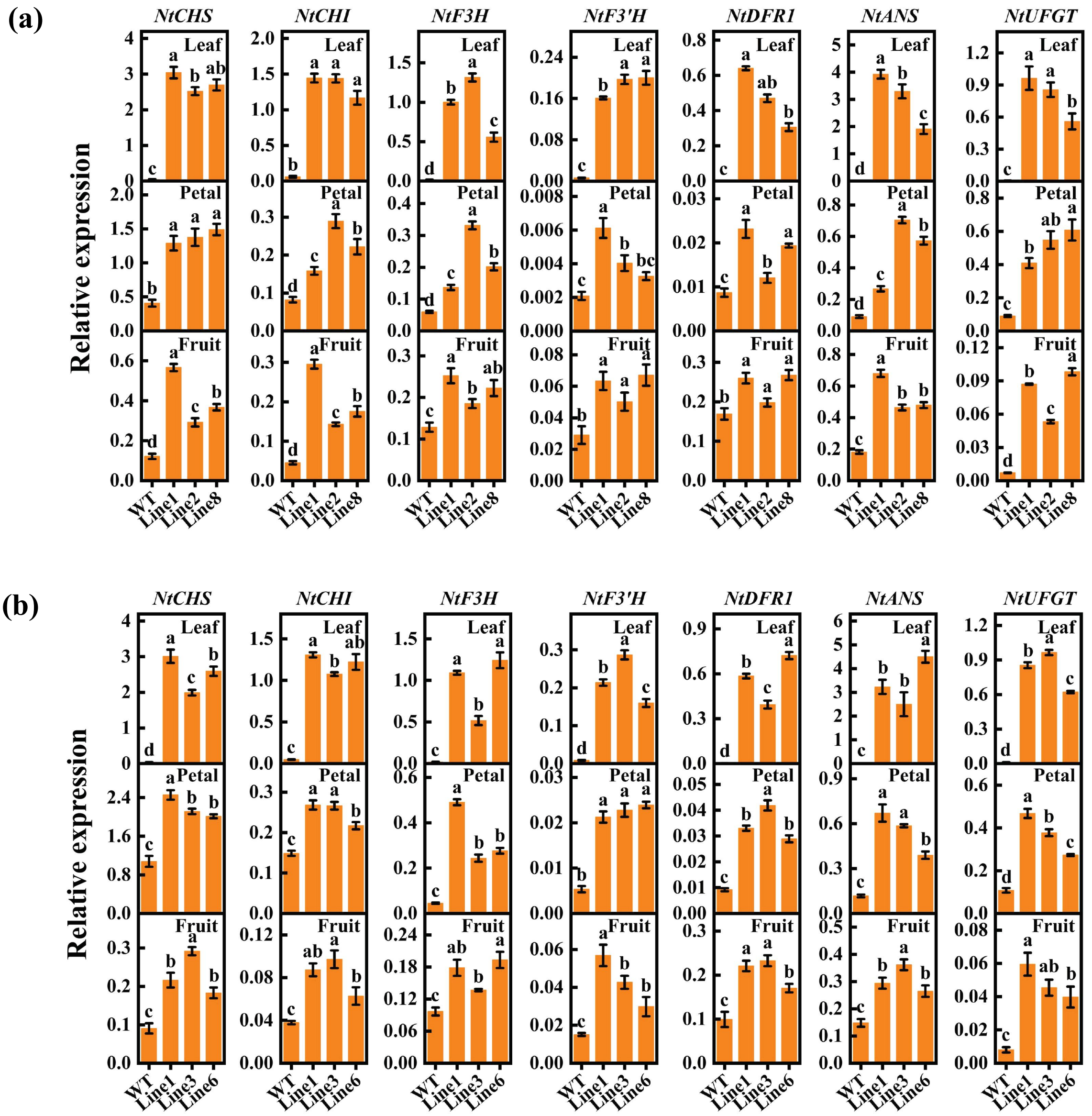

Supporting Information Fig. S9 The expression of tobacco structural genes in transgenic plants. Expression of *NtCHS*, *NtCHI*, *NtF3H*, *NtF3'H*, *NtDFR1*, *NtANS* and *NtUFGT* in leaves, flowers and fruits (pericarps and seeds) of wild-type (WT) and transgenic plants of *PpBBX32* **(a)** and *PpZAT5* **(b)**. Averaged data (± SE) from three independent biological replicates are shown. The statistical analysis was performed using one-way analysis of variance (ANOVA) testing and same letter indicates no significant difference ($P < 0.05$). ANS, anthocyanidin synthase/leucoanthocyanidin dioxygenase; CHI, chalcone isomerase; CHS, chalcone synthase; DFR, dihydroflavonol 4-reductase; F3H, flavanone 3-hydroxylase; F3'H, flavanoid 3'-hydroxylase; UFGT, (UDP)-glucose:flavonoid-3-*O*-glycosyltransferase.

(a)

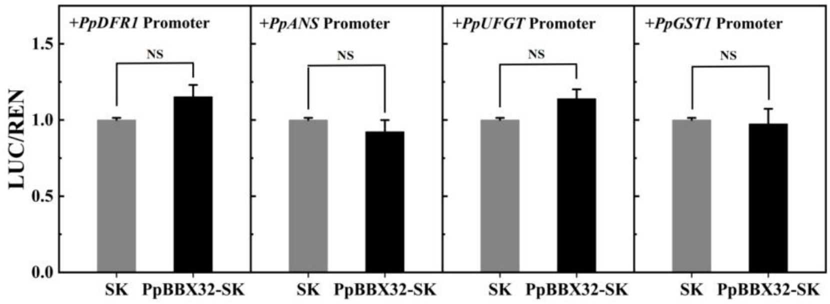

(b)

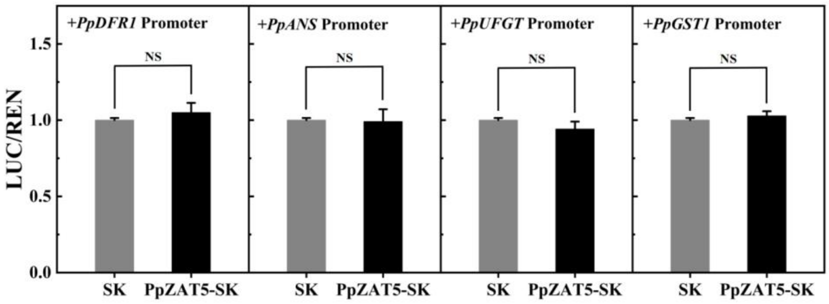

Supporting Information Fig. S10 Effects of PpBBX32 and PpZAT5 on the promoter activity of anthocyanin biosynthetic and transport related genes. The effects of PpBBX32 **(a)** and PpZAT5 **(b)** on the promoter activity of *PpDFR1*, *PpANS*, *PpUFGT* and *PpGST1* as measured by dual-luciferase (LUC) assays. Averaged data (± SE) from three independent biological replicates are shown. The statistical analysis was performed using one-way analysis of variance (ANOVA) testing. NS indicates non-significant differences at P = 0.05. ANS, anthocyanidin synthase/ leucoanthocyanidin dioxygenase; DFR, dihydroflavonol 4-reductase; GST, glutathione *S*-transferase; REN, renilla; UFGT, (UDP)-glucose:flavonoid-3-*O*-glycosyltransferase.

(a)

(b)

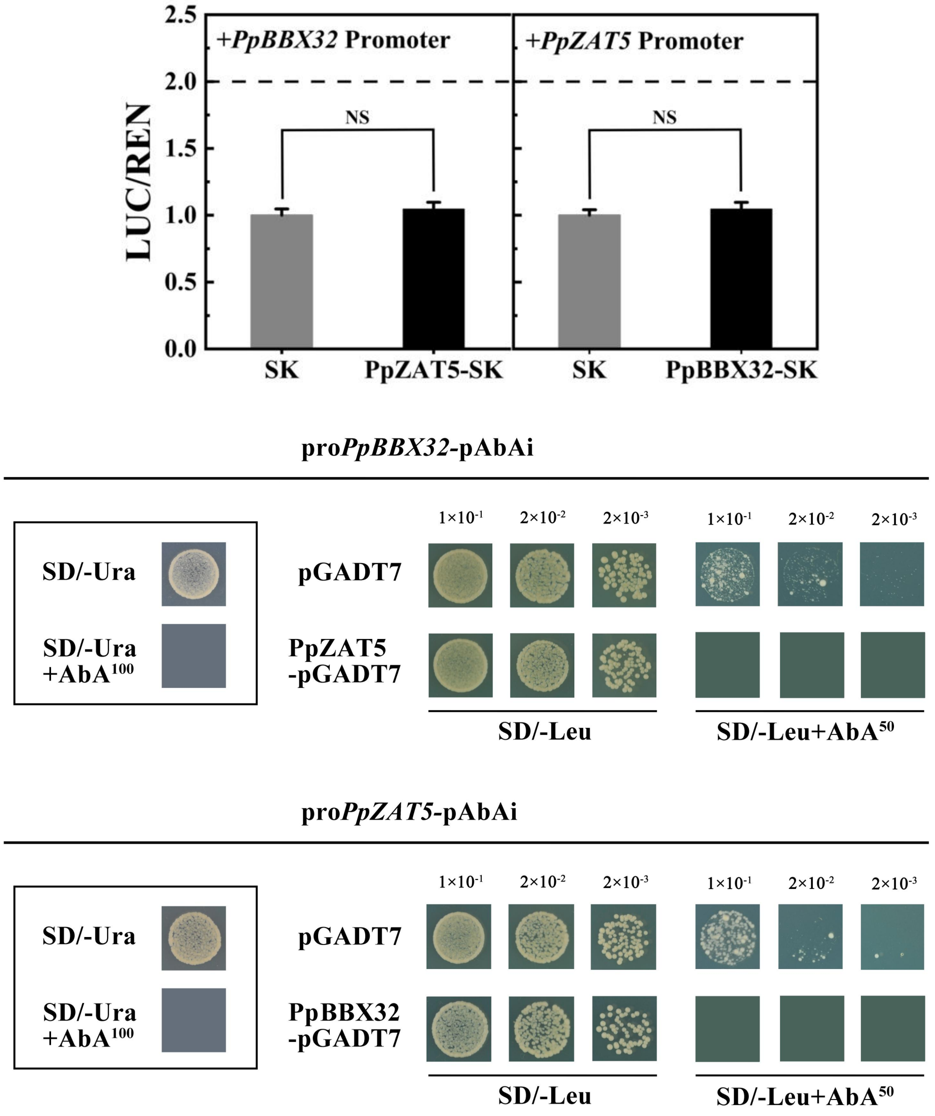

Supporting Information Fig. S11 Protein-DNA interactions between PpBBX32 and PpZAT5 reciprocally. (a) Dual-luciferase (LUC) assay. **(b)** Yeast one-hybrid (Y1H) assay. Averaged data (± SE) from three independent biological replicates are shown. The statistical analysis was performed using one-way analysis of variance (ANOVA) testing. NS indicates non-significant differences at *P* = 0.05. AbA, aureobasidin A; REN, renilla; SD, synthetic dextrose.

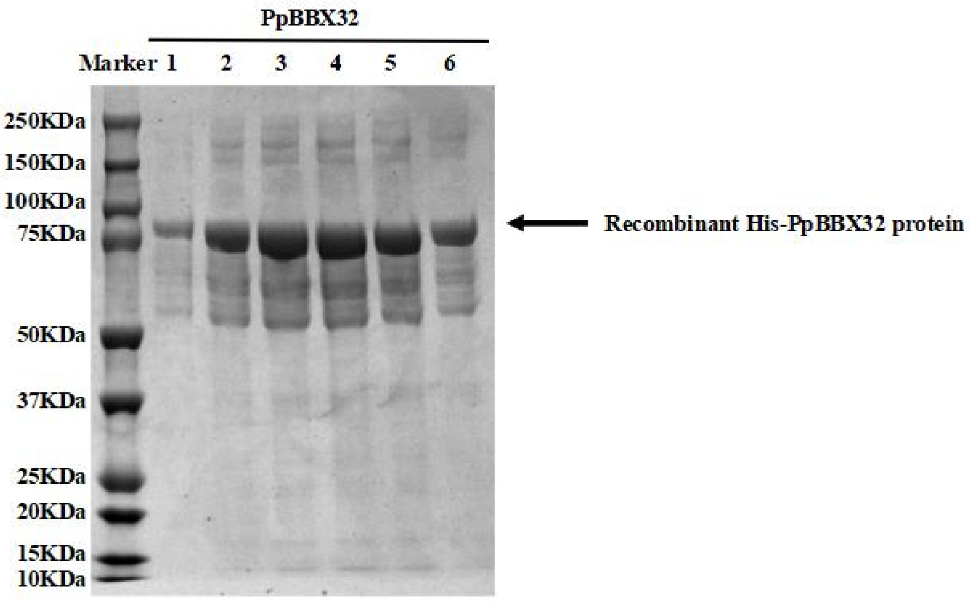

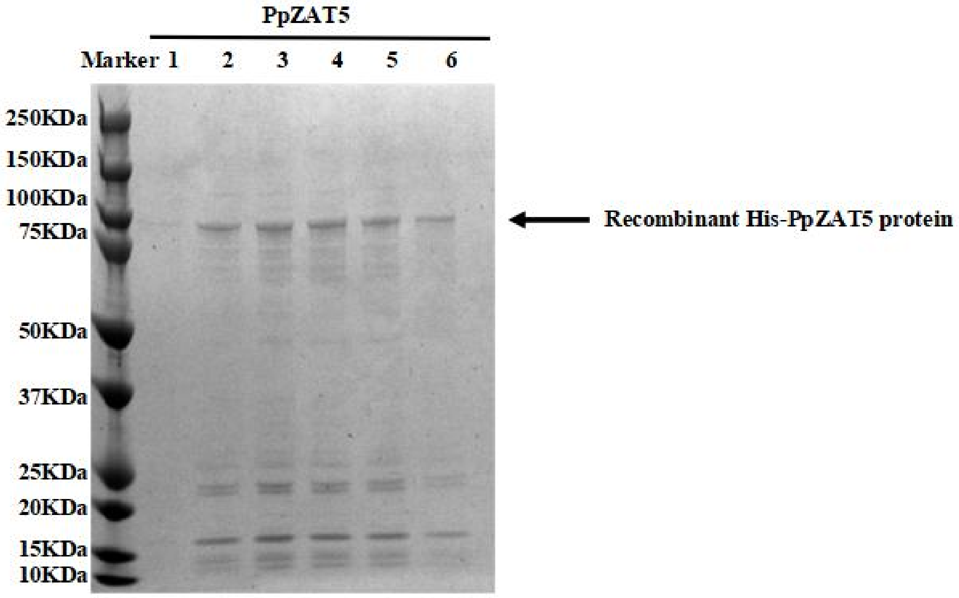

Supporting Information Fig. S12 SDS-PAGE analysis of PpBBX32 and PpZAT5.
Lanes 1-6 refer to the first six tubes of protein collected following elution.

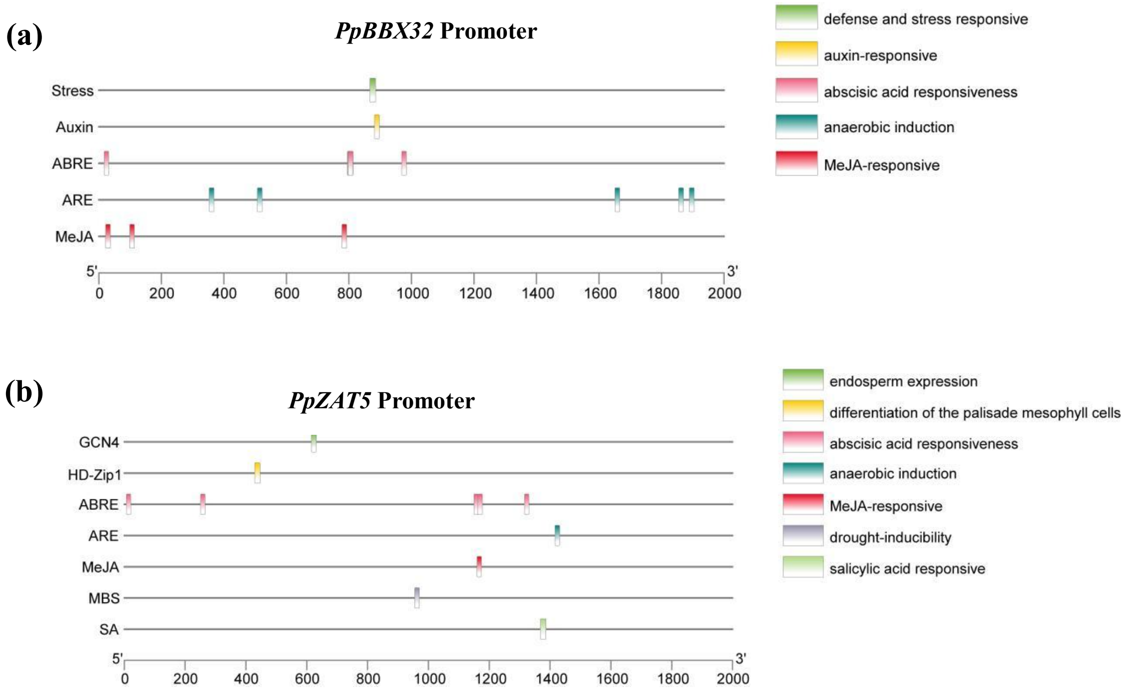

Supporting Information Fig. S13 *In silico* analysis of *cis*-acting elements in *PpBBX32* (a) and *PpZAT5* (b) promoters. The analysis was performed using PlantCARE database (http://bioinformatics.psb.ugent.be/webtools/plantcare/html/).

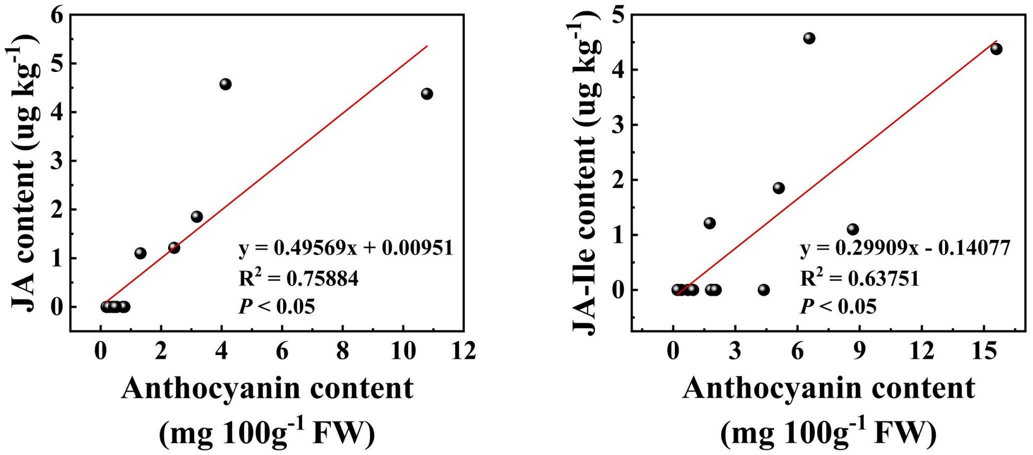

Supporting Information Fig. S14 Correlation between endogenous jasmonate (JA) / jasmonoyl-isoleucine (JA-Ile) content and anthocyanin content in the outer flesh near the peel (OF) tissues. ‘Zhonghuashoutao’ (‘ZHST’) and ‘Dongxuemi’ (‘DXM’) stored at 16°C for 30 d, OF of ‘ZHST’ stored at different temperatures for 45 d and different fruit tissues, i.e., peel (P), OF and inner flesh around the stone (IF), of ‘ZHST’ at 0 d, were used. FW, fresh weight.

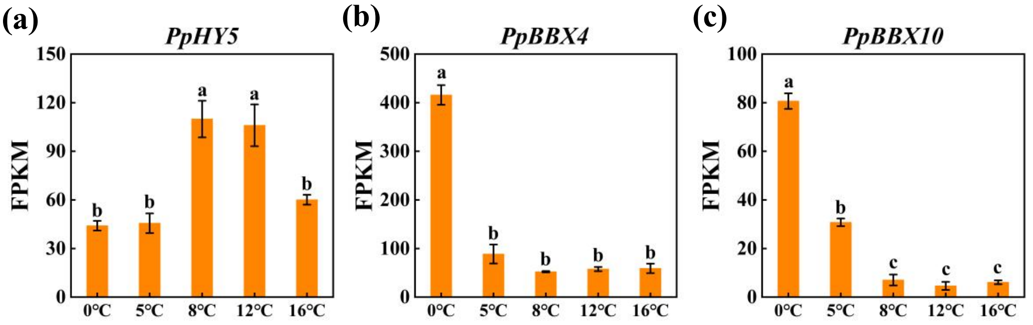

Supporting Information Fig. S15 Expression of *PpHY5* (a), *PpBBX4* (b), and *PpBBX10* (c) in the outer flesh near the peel (OF) of 'Zhonghuashoutao' ('ZHST') peach fruit. Expression data came from RNA-seq results (Zhu *et al.*, 2020). FPKM, expected number of fragments per kilobase of transcript sequence per millions base pairs sequenced. Averaged data (± SE) from three independent biological replicates are shown. The statistical analysis was performed using one-way analysis of variance (ANOVA) testing and same letter indicates no significant difference ($P < 0.05$).
