# Supplementary material for: PpBBX32 and PpZAT5 modulate temperature-dependent and tissue-specific anthocyanin accumulation in peach fruit
Source: Hortic Res. 2024 Jul 30;11(10):uhae212. doi: 10.1093/hr/uhae212 (PMC11462610; doi:10.1093/hr/uhae212)
Supplement: Web_Material_uhae212 [file web_material_uhae212.zip › Suppl Tables-HR.pdf]

Supporting Information Table S1. Summary of the transcriptome sequencing data and read mapping of 'Zhonghuashoutao' ('ZHST') and 'Dongxuemi' ('DXM') peach libraries

ZHST & DXM							
Sample name	Raw reads	Clean reads	Q20(%)	Q30(%)	Total mapped	Multiple mapped	Uniquely mapped
ZHST0d-1	51981774	50068462	98.36	94.88	48791695 (97.45%)	1343931 (2.68%)	47447764 (94.77%)
ZHST0d-2	45590510	43816120	98.43	95.11	42799239 (97.68%)	976479 (2.23%)	41822760 (95.45%)
ZHST0d-3	44734722	43067762	98.35	95	41886870 (97.26%)	1004951 (2.33%)	40881919 (94.92%)
ZHST15d-1	48294248	47092116	97.91	93.53	44601981 (94.71%)	970883 (2.06%)	43631098 (92.65%)
ZHST15d-2	46030358	45682486	98.33	94.75	43637706 (95.52%)	936002 (2.05%)	42701704 (93.48%)
ZHST15d-3	53733540	51273992	98.36	95.01	49174930 (95.91%)	1074813 (2.1%)	48100117 (93.81%)
ZHST30d-1	45635826	44111570	98.4	95.05	43074611 (97.65%)	814479 (1.85%)	42260132 (95.8%)
ZHST30d-2	46243140	44621422	98.42	95.06	42965953 (96.29%)	812423 (1.82%)	42153530 (94.47%)
ZHST30d-3	46424570	45413276	97.71	93.53	42110072 (92.73%)	786011 (1.73%)	41324061 (91.0%)
DXM0d-1	42529112	41014408	98.65	95.54	39207438 (95.59%)	764930 (1.87%)	38442508 (93.73%)
DXM0d-2	46447832	43635402	98.4	95.05	42535513 (97.48%)	818849 (1.88%)	41716664 (95.6%)
DXM0d-3	43495736	40893786	98.35	94.85	38716750 (94.68%)	690998 (1.69%)	38025752 (92.99%)
DXM15d-1	45824844	44026278	98.45	95.1	41266673 (93.73%)	745589 (1.69%)	40521084 (92.04%)
DXM15d-2	47288756	44271940	98.02	93.74	42573316 (96.16%)	733117 (1.66%)	41840199 (94.51%)
DXM15d-3	45655188	43340698	98.35	94.85	40291115 (92.96%)	692166 (1.6%)	39598949 (91.37%)
DXM30d-1	48024300	46461272	98.48	95.19	45569447 (98.08%)	764972 (1.65%)	44804475 (96.43%)
DXM30d-2	47176474	45489170	98.39	94.98	43913699 (96.54%)	722311 (1.59%)	43191388 (94.95%)
DXM30d-3	46592940	44326690	98.39	94.99	42962231 (96.92%)	740474 (1.67%)	42221757 (95.25%)

Supporting Information Table S2. Summary of the transcriptome sequencing data and read mapping the peel (P), the outer flesh near the peel (OF) and the inner flesh around the stone (IF) of 'Zhonghuashoutao' ('ZHST') peach fruit libraries

ZHST							
Sample name	Raw reads	Clean reads	Q20(%)	Q30(%)	Total mapped	Multiple mapped	Uniquely mapped
P-1	47808500	46442148	97.67	93.34	40900943 (88.07%)	1002745 (2.16%)	39898198 (85.91%)
P-2	43694398	41612642	97.56	93.1	40030790 (96.2%)	826412 (1.99%)	39204378 (94.21%)
P-3	45475220	43635336	97.57	93.1	39668280 (90.91%)	876644 (2.01%)	38791636 (88.9%)
OF-1	47279792	45329942	97.4	92.75	40203433 (88.69%)	878309 (1.94%)	39325124 (86.75%)
OF-2	42334576	40395052	97.45	92.82	36473488 (90.29%)	711827 (1.76%)	35761661 (88.53%)
OF-3	55066764	52610598	97.58	93.13	44132499 (83.89%)	1371196 (2.61%)	42761303 (81.28%)
IF-1	46944454	44811438	97.48	92.9	39436517 (88.01%)	964950 (2.15%)	38471567 (85.85%)
IF-2	46553558	44787158	97.68	93.36	41389111 (92.41%)	932928 (2.08%)	40456183 (90.33%)
IF-3	45524150	43712412	97.06	91.97	41212262 (94.28%)	796424 (1.82%)	40415838 (92.46%)

Supporting Information Table S3. List of 26 differentially expressed transcription factors (TFs) from the Purple module. TFs were listed in descending order by the correlation between gene expression and anthocyanin content. Two candidate transcription factors further analysed in this study were highlighted in red.

Number	Gene ID	Gene name	Function	Gene significance		Gene significance	
				Anthocyanin	*P*	Purple module	*P*
1	Prupe.4G138200	LBD4	LOB domain-containing protein 4	0.9974	0.0002	0.9931	0.0007
2	Prupe.6G159200	HEC3	Transcription factor HEC3-related	0.9972	0.0002	0.9929	0.0007
3	Prupe.7G149700	HB40	Homeobox protein 40	0.9954	0.0004	0.9834	0.0026
4	Prupe.7G064600	B3	B3 DNA binding domain	0.9925	0.0008	0.9787	0.0037
**5**	**Prupe.3G020100**	**BBX32**	**B-box type zinc finger-containing protein**	**0.9896**	**0.0014**	**0.9973**	**0.0002**
6	Prupe.7G155000	RL1	RAD-like 1	0.9881	0.0016	0.9972	0.0002
7	Prupe.3G267900	MIF2	mini zinc finger 2	0.9872	0.0017	0.9656	0.0076
8	Prupe.1G531100	SVP	MADS-box protein	0.9853	0.0021	0.9819	0.0029
9	Prupe.4G242700	NF-YB3	nuclear factor Y, subunit B3	0.9844	0.0023	0.9913	0.001
10	Prupe.2G182800	GBF3	G-box binding factor 3	-0.9824	0.0028	-0.9356	0.0195
11	Prupe.1G223500	SBP	SBP domain	0.981	0.0031	0.9842	0.0024
12	Prupe.1G271400	HB13	Homeobox-leucine zipper protein family	0.9778	0.004	0.9422	0.0166
**13**	**Prupe.7G125800**	**ZAT5**	**C2H2-type zinc finger family protein**	**0.9756**	**0.0046**	**0.9517**	**0.0127**
14	Prupe.3G170600	AGL1	K-box region and MADS-box transcription factor family protein	0.9654	0.0077	0.9903	0.0012
15	Prupe.5G075800	YABBY	YABBY protein	0.9553	0.0113	0.9797	0.0035

16	Prupe.1G447100	MEKHLA	MEKHLA domain	0.9539	0.0118	0.9813	0.0031
17	Prupe.2G156900	ANL2	lipid-binding START domain-containing protein	0.9520	0.0136	0.9323	0.0032
18	Prupe.6G091100	RAP2.7	related to AP2.7	-0.9515	0.0151	0.9021	0.0362
19	Prupe.7G183300	WOX13	WUSCHEL related homeobox 13	0.9455	0.017	0.9717	0.0057
20	Prupe.5G093200	HSFB2b	winged-helix DNA-binding transcription factor family protein	-0.9312	0.0247	-0.9356	0.0195
21	Prupe.1G531600	AGL22	K-box region and MADS-box transcription factor family protein	-0.9206	0.0261	-0.9525	0.0122
22	Prupe.3G283200	HD-ZIP-1	homeobox 1	-0.9185	0.0289	-0.9274	0.0231
23	Prupe.3G155900	STO	B-box zinc finger family protein	-0.9057	0.0379	-0.9477	0.0143
24	Prupe.4G222300	ERF7	ethylene response factor 7	-0.8985	0.0392	-0.9358	0.0193
25	Prupe.2G004000	HAT14	homeobox from Arabidopsis thaliana	-0.8969	0.0411	-0.9495	0.0135
26	Prupe.6G182200	RAP2.4	related to AP2.4	-0.8837	0.0467	-0.9068	0.0337

Supporting Information Table S4. List of the top 50 differentially expressed genes (DEGs) most related to anthocyanin phenotype as revealed by transcriptome analysis of three fruit tissue types of 'Zhonghuashoutao' ('ZHST'). The anthocyanin biosynthetic genes, *BBX* family genes and *C2H2* zinc finger family genes were highlighted in red, purple and blue, respectively.

No.	GeneID	Gene name	Gene correlation	
			Anthocyanin	*P*
1	Prupe.4G006200	NA	0.951663492	7.80E-05
**2**	**Prupe.3G020100**	**BBX32**	**0.946181983**	**0.000112938**
3	Prupe.3G240000	RAVL1	0.943279968	0.000135338
4	Prupe.4G059600	SAG12	0.941713466	0.000148647
5	Prupe.2G326300	4CL6	0.939745024	0.000166645
6	Prupe.5G131300	GUN1	0.935519184	0.000210377
7	Prupe.4G033400	PBP1	0.934373452	0.000223499
**8**	**Prupe.8G142400**	**ZAT10**	**0.932970006**	**0.000240343**
9	Prupe.3G205100	FLA7	0.931847648	0.000254441
10	Prupe.1G002900	CHS1	0.93149885	0.000258938
11	Prupe.I005800	CHS3	0.930708623	0.000269332
12	Prupe.6G235400	PAL2	0.930655659	0.000270039
13	Prupe.3G013600	GST1	0.92964537	0.000283776
**14**	**Prupe.7G125800**	**ZAT5**	**0.928638554**	**0.000297946**
15	Prupe.6G058300	AMT13	0.928300909	0.000302807
16	Prupe.1G376400	DFR1	0.927365735	0.000316562
17	Prupe.5G106800	GUN	0.926727501	0.000326197
18	Prupe.8G155500	BCAT2	0.926258598	0.000333406
19	Prupe.1G003000	CHS2	0.925981816	0.000337713
20	Prupe.1G264900	GSTUH	0.925930989	0.000338508
21	Prupe.2G324700	UFGT	0.925562205	0.000344317
22	Prupe.8G201000	XTH2	0.924883195	0.000355194
23	Prupe.5G203600	F3'H	0.924003806	0.000369638
**24**	**Prupe.5G201400**	**ZAT4**	**0.923712275**	**0.000374515**
25	Prupe.5G081600	ACR8	0.923056019	0.00038566
26	Prupe.4G059100	DSEL	0.922992806	0.000386746
27	Prupe.6G329500	ATL6	0.922048177	0.000403225
**28**	**Prupe.4G192700**	**BBX26**	**0.919706872**	**0.000446183**

29	Prupe.3G286200	TKPR1	0.919333477	0.000453318
30	Prupe.3G184800	UFOG3	0.919060755	0.000458579
31	Prupe.I005700	CHS4	0.919002852	0.000459702
32	Prupe.1G340500	MYBF	0.917718253	0.000485105
33	Prupe.3G163100	MYB10.1	0.917427772	0.000490983
**34**	**Prupe.4G033600**	**BBX28**	**0.917346562**	**0.000492635**
**35**	**Prupe.1G424300**	**ZAT10**	**0.916631782**	**0.000507344**
36	Prupe.3G287200	PGLR4	0.916321477	0.000513824
37	Prupe.6G040400	C4H	0.916304547	0.00051418
38	Prupe.8G163200	TLP1	0.913460698	0.000576324
39	Prupe.1G425400	SBT17	0.913127777	0.000583926
40	Prupe.5G068400	WTR44	0.912624097	0.000595561
41	Prupe.4G215000	GGR	0.912472975	0.000599083
42	Prupe.6G283700	FRI3	0.91229497	0.00060325
43	Prupe.8G256900	GSTF	0.911951177	0.000611356
44	Prupe.5G068200	1MMP	0.911882449	0.000612986
45	Prupe.8G103100	U496A	0.910219786	0.00065334
**46**	**Prupe.6G084100**	**ZAT12**	**0.908267878**	**0.00070304**
47	Prupe.3G241700	TKPR1	0.908104105	0.000707327
48	Prupe.5G086700	LDOX/ANS	0.907628147	0.000719888
49	Prupe.1G375700	JMT	0.905476779	0.000778612
50	Prupe.1G112100	SFR2	0.904909749	0.000794629

Supporting Information Table S5. Predicted plant hormone related *cis*-acting elements in the promoter regions of *PpBBX32* and *PpZAT5*

***Cis*-Element Name**	***Cis*-Element Sequence (5'-3')**	**Function**
ABRE	ACGTG	*cis*-acting element involved in the abscisic acid responsiveness
CGTCA-motif	CGTCA	*cis*-acting regulatory element involved in the MeJA-responsiveness
TGACG-motif	TGACG	*cis*-acting regulatory element involved in the MeJA-responsiveness

Supporting Information Table S6. **Primers used for RT-qPCR analysis of anthocyanin related genes in peach and tobacco**

Gene name	Forward primer (5′ to 3′)	Reverse primer (5′ to 3′)	GeneID
PpCHS1	AACCATCCTTCCCGACAGCGAT	CAGAGATACCCAAAGGTTGGAAGGC	Prupe.1G002900
PpCHI1	ACACAGGTGACAACGATACTGC	GACCTCAAGGAACTTCTCAATGG	Prupe.2G225200
PpF3H	TTGTGGAGGCTTGTGAGGATT	CGAGGGCAGAGCGAAGAACT	Prupe.7G168300
PpF3'H	CCCAACTTGACCTACCTCCA	CTTTGGGATGTGGAAGCTGT	Prupe.5G203600
PpDFR1	CGCCTCCAAGACTCTAGCTG	CCAGTGAGTGGGGAAAGTCC	Prupe.1G376400
PpANS	TGCCAAGTGTGTTCCAAATTCC	GGCTCACAGAAAACTGCCCAT	Prupe.5G086700
PpUFGT	CTGCCTCTCCCAACACTCTCTT	TCAGCCACATCAAACACCTTTATG	Prupe.2G324700
PpGST1	GTACTTCAACTTCTGGTGCTGC	AGCTGCTCTTTGATAATCTTTCCTC	Prupe.3G013600
PpMYB10.1	CAGGAAGGACAGCGAATGATG	TCGGGGTTGAGGTCTTATTACG	Prupe.3G163100
PpbHLH3	TTGTTCAGCGTTCCGTTCCT	GCGCTGAGCTCATCTTGTGG	Prupe.8G242100
PpWD40	CCAGCCTGATACCCCTTTGCTT	CGGCGAACGGATATCCAAAATC	Prupe.2G319500
PpBBX32	CAGTCCTCTTCCTCGGCTTG	CGGAATATTCGACAACTTGCCG	Prupe.3G020100
PpZAT5	GTTCACAACAATAAGGCTTCAGCT	CCTTGTTGGGTTTCTTGTGACTG	Prupe.7G125800
PpTEF2 (Actin)	GGTGTGACGATGAAGAGTGATG	TGAAGGAGAGGGAAGGTGAAAG	JQ732180
NtCHS	TTGTTCGAGCTTGTCTCTGC	AGCCCAGGAACATCTTTGAG	AF311783
NtCHI	GTCAGGCCATTGAAAAGCTC	CTAATCGTCAATGCCCCAAC	AB213651
NtF3H	CAAGGCATGTGTGGATATGG	TGTGTCGTTTCAGTCCAAGG	AB289450
NtF3'H	AGGCTCAACACTTCTCGT	CATCAACTTTGGGCTTCT	AB289449
NtDFR1	AACCAACAGTCAGGGGAATG	TTGGACATCGACAGTTCCAG	EF421429
NtANS	TGGCGTTGAAGCTCATACTG	GGAATTAGGCACACACTTTGC	AB289447
NtUFGT	GAGTGCATTGGATGCCTTTT	CCAGCTCCATTAGGTCCTTG	FG627024
NtAn2	GAAGAAAGGTGCATGGACTG	TCTGCAGCTCTTTCTGCATC	FJ472647
NtAn1a	ACCATTCTCGAACACCGAAG	TGCTAGGGCACAATGTGAAG	HQ589208
NtAn1b	CTTGAACACTTCCTCAAACCGA	TGCTAGGGCACAATGTGAAG	HQ589209
NtACT	AATGGAACTGGAATGGTCAAGGC	TGCCAGATCTTCTCCATGTCATCCCA	AJ421411

Supporting Information Table S7. Gene IDs for plant BBXs and ZATs used in phylogenetic analysis and sequence alignment

Gene name	Species	Gene ID
AtBBX4	*Arabidopsis thaliana*	AT2G24790
AtBBX21	*Arabidopsis thaliana*	AT1G75540
AtBBX22	*Arabidopsis thaliana*	AT1G78600
AtBBX23	*Arabidopsis thaliana*	AT4G10240
AtBBX24	*Arabidopsis thaliana*	AT1G06040
AtBBX25	*Arabidopsis thaliana*	AT2G31380
AtBBX31	*Arabidopsis thaliana*	AT3G21890
AtBBX32	*Arabidopsis thaliana*	AT3G21150
MaBBX20	*Muscari spp.*	MW160173
MaBBX51	*Muscari spp.*	MW160172
MdBBX1	*Malus domestica*	MDP0000259614
MdBBX20	*Malus domestica*	MDP0000177126
MdBBX22	*Malus domestica*	MDP0000298804
MdBBX33	*Malus domestica*	MDP0000697407
MdBBX37	*Malus domestica*	MDP0000157816
MdCOL4	*Malus domestica*	HM122534.1
OsBBX14	*Oryza sativa*	Os06g0713000
PpBBX32	*Prunus persica*	Prupe.3G020100
PpyBBX16	*Pyrus pyrifolia*	Pbr020473.1
PpyBBX18	*Pyrus pyrifolia*	Pbr005884.1
PpyBBX21	*Pyrus pyrifolia*	Pbr034751.1
SlBBX20	*Solanum lycopersicum*	Solyc01g110180
VcBBX	*Vaccinium corymbosum*	KX300037.1
AtZAT1	*Arabidopsis thaliana*	AT2G46800
AtZAT2	*Arabidopsis thaliana*	AT2G17180
AtZAT3	*Arabidopsis thaliana*	AT4G35280
AtZAT4	*Arabidopsis thaliana*	AT2G45120
AtZAT5	*Arabidopsis thaliana*	AT2G28200
AtZAT6	*Arabidopsis thaliana*	AT5G04340
AtZAT7	*Arabidopsis thaliana*	AT3G46090
AtZAT8	*Arabidopsis thaliana*	AT3G46080
AtZAT9	*Arabidopsis thaliana*	AT3G60580
AtZAT10	*Arabidopsis thaliana*	AT1G27730
AtZAT11	*Arabidopsis thaliana*	AT2G37430
AtZAT12	*Arabidopsis thaliana*	AT5G59820
AtZAT18	*Arabidopsis thaliana*	AT3G53600
MdZAT5	*Malus domestica*	MD03G1128800
PpZAT5	*Prunus persica*	Prupe.7G125800
PpyZAT5	*Pyrus pyrifolia*	XP_009356940.1
SlZF2	*Solanum lycopersicum*	ADZ15317

Supporting Information Table S8. Subcloning primers and electrophoretic mobility shift assay (EMSA) probes used in this study. Nucleotides in lower case are identical to the sequence of the vector.

Name	Sequence (5′ - 3′)	Purpose
PpBBX32-SAK-FP	actagtggatccaaagaattcATGAAAGCTAGGGTTTGCGAGC	Subclone to pSAK277 Vector
PpBBX32-SAK-RP	gactctagaagtactctcgagTCAACACTCGGCCCACCC	
PpZAT5-SAK-FP	actagtggatccaaagaattcATGATGATCATCAAAGGCAAACG	
PpZAT5-SAK-RP	gactctagaagtactctcgagTTAGTAATGGCAACCCACCAAA	
PpMYB10.1-SAK-FP	actagtggatccaaagaattcATGGAGGGCTATAACTTGGGTG	
PpMYB10.1-SAK-RP	gactctagaagtactctcgagTTAATGATTCCAAAAGTCCACGTT	
PpbHLH3-SAK-FP	actagtggatccaaagaattcATGCAGCTAGAGATGTCCGAGG	
PpbHLH3-SAK-RP	gactctagaagtactctcgagCTAGGAATCAGATTGGGGAATTATTT	
PpBBX32-pTRV2-FP	agaaggcctccatggggatccGCCAAAGGAATCAGTGCTAGAGA	Subclone to pTRV2 Vector
PpBBX32-pTRV2-RP	gggacatgcccgggcctcgagATGAAAGCTAGGGTTTGCGAGC	
PpZAT5-pTRV2-FP	agaaggcctccatggggatccTCCCGATTCGATATTTGTAACGA	
PpZAT5-pTRV2-RP	gggacatgcccgggcctcgagTTTGATTCTTTTGGCTCAAGGAA	
proPpBBX32-LUC-FP	ggcgaattgggtaccgggcccATCATTCCAATCACGCCAAACT	Subclone to pGreenII 0800-LUC Vector
proPpBBX32-LUC-RP	cgctctagaactagtggatccGGTGGAGAAGGCGAGAGAGC	
proPpZAT5-LUC-FP	ggcgaattgggtaccgggcccCCATGTTATGTCACCAACCAAAA	
proPpZAT5-LUC-RP	cgctctagaactagtggatccCTGGCTCTGATCCTTATAACCCA	
proPpMYB10.1^{-2074}-LUC-FP	ggcgaattgggtaccgggcccGTCGAACCTAATTGCCACATAAATT	
proPpMYB10.1^{-1728}-LUC-FP	ggcgaattgggtaccgggcccCGTATTAATTGAAAAAAATAATTTATACAAG	
proPpMYB10.1^{-1031}-LUC-FP	ggcgaattgggtaccgggcccCCATAAATATTGTTGTCAATCCACTG	
proPpMYB10.1^{-806}-LUC-FP	ggcgaattgggtaccgggcccTAAAAGGTTACACGATGCTGCATT	

proPpMYB10.1^{-672}-LUC-FP	ggcgaattgggtaccgggcccACAGAGATAACGGTGATTGGAGG	
proPpMYB10.1^{-563}-LUC-FP	ggcgaattgggtaccgggcccGTGAAAACCAGAACTTGTAACTAATCCG	
proPpMYB10.1^{-379}-LUC-FP	ggcgaattgggtaccgggcccTATCATATGTTTAGCCTATAACTAATGCTTAA	
proPpMYB10.1^{-270}-LUC-FP	ggcgaattgggtaccgggcccGTTATCAAGCCGTACAAGAAAACG	
proPpMYB10.1^{-216}-LUC-FP	ggcgaattgggtaccgggcccGATGGAGAACCGTACGTAGCCA	
proPpMYB10.1-LUC-RP	cgctctagaactagtggatccCTTCTTGTTGGCCAGCGTTG	
proPpDFR1-LUC-FP	ggcgaattgggtaccgggcccTGGGATTAGAGCACCTTCACCC	
proPpDFR1-LUC-RP	cgctctagaactagtggatccATTTGAATCAAATCAAGTATGTACTGCT	
proPpANS-LUC-FP	ggcgaattgggtaccgggcccTTATTCTTATCTCCATCCCTGTTAAGC	
proPpANS-LUC-RP	cgctctagaactagtggatccTTTGGCAGCCGGCTCTTC	
proPpUFGT-LUC-FP	ggcgaattgggtaccgggcccTACAACCTGAAATTCTTCCTTGGC	
proPpUFGT-LUC-RP	cgctctagaactagtggatccATATGTATGAGCTAATAAGACTAATTGGAGTG	
proPpGST1-LUC-FP	ggcgaattgggtaccgggcccCAGTCAACATACTTTCGGGCTAAG	
proPpGST1-LUC-RP	cgctctagaactagtggatccCTTGTTATATTAATATCTCTCTCACTCCTTAAC	
PpBBX32-SK-FP	cgctctagaactagtggatccATGAAAGCTAGGGTTTGCGAGC	Subclone to pGreenII 62-SK Vector
PpBBX32-SK-RP	cagcgaattggtaccgggcccTCAACACTCGGCCCACCC	
PpZAT5-SK-FP	cgctctagaactagtggatccATGATGATCATCAAAGGCAAACG	
PpZAT5-SK-RP	cagcgaattggtaccgggcccTTAGTAATGGCAACCCACCAAA	
proPpBBX32-pAbAi-FP	gaaaagcttgaattcgagctcTCCAAATATGTCATTGTGGTTTGAT	Subclone to pAbAi Vector
proPpBBX32-pAbAi-RP	atacagagcacatgcctcgagTGTGTCTGTATTTGGCTTTGCC	
proPpZAT5-pAbAi-FP	gaaaagcttgaattcgagctcCCATGTTATGTCACCAACCAAAA	
proPpZAT5-pAbAi-RP	atacagagcacatgcctcgagCTGGCTCTGATCCTTATAACCCA	
proPpMYB10.1-pAbAi-FP	gaaaagcttgaattcgagctcGTCGAACCTAATTGCCACATAAATT	
proPpMYB10.1-pAbAi-RP	atacagagcacatgcctcgagAACAGTTAGCTAAGAGTTTGTTAGTGTCAG	

PpBBX32-pGADT7-FP	gccatggaggccagtgaattcATGAAAGCTAGGGTTTGCGAGC	Subclone to pGADT7 Vector
PpBBX32-pGADT7-RP	cagctcgagctcgatggatccTCAACACTCGGCCCACCC	
PpZAT5-pGADT7-FP	gccatggaggccagtgaattcATGATGATCATCAAAGGCAAACG	
PpZAT5-pGADT7-RP	cagctcgagctcgatggatccTTAGTAATGGCAACCCACCAAA	
PpMYB10.1-pGADT7-FP	gccatggaggccagtgaattcATGGAGGGCTATAACTTGGGTG	
PpMYB10.1-pGADT7-RP	cagctcgagctcgatggatccTTAATGATTCCAAAAGTCCACGTT	
PpBBX32-pGBKT7-FP	atggccatggaggccgaattcATGAAAGCTAGGGTTTGCGAGC	Subclone to pGBKT7 Vector
PpBBX32-pGBKT7-RP	ccgctgcaggtcgacggatccTCAACACTCGGCCCACCC	
PpZAT5-pGBKT7-FP	atggccatggaggccgaattcATGATGATCATCAAAGGCAAACG	
PpZAT5-pGBKT7-RP	ccgctgcaggtcgacggatccTTAGTAATGGCAACCCACCAAA	
PpMYB10.1-pGBKT7-FP	atggccatggaggccgaattcATGGAGGGCTATAACTTGGGTG	
PpMYB10.1-pGBKT7-RP	ccgctgcaggtcgacggatccTTAATGATTCCAAAAGTCCACGTT	
PpBBX32-pColdTF-FP	gaaggtaggcatatggagctcATGAAAGCTAGGGTTTGCGAGC	Subclone to pCold-TF Vector
PpBBX32-pColdTF-RP	ctatctagactgcaggtcgacACACTCGGCCCACCCCTC	
PpZAT5-pColdTF-FP	gaaggtaggcatatggagctcATGATGATCATCAAAGGCAAACG	
PpZAT5-pColdTF-RP	ctatctagactgcaggtcgacGTAATGGCAACCCACCAAAGG	
PpBBX32-cLUC-FP	tacgcgtcccggggcggtaccATGAAAGCTAGGGTTTGCGAGC	Subclone to pCAMBIA1300-cLUC
PpBBX32-cLUC-RP	acgaaagctctgcaggtcgacTCAACACTCGGCCCACCC	
PpZAT5-cLUC-FP	tacgcgtcccggggcggtaccATGATGATCATCAAAGGCAAACG	
PpZAT5-cLUC-RP	acgaaagctctgcaggtcgacTTAGTAATGGCAACCCACCAAA	
PpMYB10.1-cLUC-FP	tacgcgtcccggggcggtaccATGGAGGGCTATAACTTGGGTG	
PpMYB10.1-cLUC-RP	acgaaagctctgcaggtcgacTTAATGATTCCAAAAGTCCACGTT	
PpBBX32-nLUC-FP	acgggggacgagctcggtaccATGAAAGCTAGGGTTTGCGAGC	Subclone to pCAMBIA1300-nLUC
PpBBX32-nLUC-RP	cgcgtacgagatctggtcgacACACTCGGCCCACCCCTC	

PpZAT5-nLUC-FP	acgggggacgagctcggtaccATGATGATCATCAAAGGCAAACG	
PpZAT5-nLUC-RP	cgcgtacgagatctggtcgacGTAATGGCAACCCACCAAAGG	
PpMYB10.1-nLUC-FP	acgggggacgagctcggtaccATGGAGGGCTATAACTTGGGTG	
PpMYB10.1-nLUC-RP	cgcgtacgagatctggtcgacATGATTCCAAAAGTCCACGTTAAA	
proPpMYB10.1-G-box1&2-FP	CAGCTGTGATACGTGAACCACACGTGCTGACACTA	Probe for EMSA
proPpMYB10.1-G-box1&2-RP	TAGTGTCAGCACGTGTGGTTCACGTATCACAGCTG	
proPpMYB10.1-G-box1&2-mut-FP	CAGCTGTGACGTTCAAACCATGTCCACTGACACTA	
proPpMYB10.1-G-box1&2-mut-RP	TAGTGTCAGTGGACATGGTTTGAACGTCACAGCTG	
proPpMYB10.1-G-box1-mut-FP	CAGCTGTGATACGTGAACCATGTCCACTGACACTA	
proPpMYB10.1-G-box1-mut-RP	TAGTGTCAGTGGACATGGTTCACGTATCACAGCTG	
proPpMYB10.1-G-box2-mut-FP	CAGCTGTGACGTTCAAACCACACGTGCTGACACTA	
proPpMYB10.1-G-box2-mut-RP	TAGTGTCAGCACGTGTGGTTTGAACGTCACAGCTG	
proPpMYB10.1-biotin-FP	TCTTAGCTAACTGTTACTGTTATCAAG	
proPpMYB10.1-biotin-RP	CTTGATAACAGTAACAGTTAGCTAAGA	
proPpMYB10.1-mutant-FP	TCTTAGCTAGTTACCCTAGTTATCAAG	
proPpMYB10.1-mutant-RP	CTTGATAACTAGGGTAACTAGCTAAGA	
